# Endoribonuclease YbeY Is Essential for RNA Processing and Virulence in Pseudomonas aeruginosa

**DOI:** 10.1128/mBio.00659-20

**Published:** 2020-06-30

**Authors:** Yushan Xia, Yuding Weng, Congjuan Xu, Dan Wang, Xiaolei Pan, Zhenyang Tian, Bin Xia, Haozhou Li, Ronghao Chen, Chang Liu, Yongxin Jin, Fang Bai, Zhihui Cheng, Oscar P. Kuipers, Weihui Wu

**Affiliations:** a State Key Laboratory of Medicinal Chemical Biology, Key Laboratory of Molecular Microbiology and Technology of the Ministry of Education, Department of Microbiology, College of Life Sciences, Nankai University, Tianjin, China; b Department of Molecular Genetics, Groningen Biomolecular Sciences and Biotechnology Institute, University of Groningen, Groningen, Netherlands; Mass. General Hospital/Harvard Medical School; Harvard Medical School

**Keywords:** endoribonuclease, *Pseudomonas aeruginosa*, ReaL, RpoS, YbeY

## Abstract

Posttranscriptional regulation plays an essential role in the quick adaptation of pathogenic bacteria to host environments, and RNases play key roles in this process by modifying small RNAs and mRNAs. We find that the Pseudomonas aeruginosa endonuclease YbeY is required for rRNA processing and the bacterial virulence in a murine acute pneumonia model. Transcriptomic analyses reveal that knocking out the *ybeY* gene results in downregulation of oxidative stress response genes, including the catalase genes *katA* and *katB*. Consistently, the *ybeY* mutant is more susceptible to H_2_O_2_ and neutrophil-mediated killing. Overexpression of *katA* restores the bacterial tolerance to H_2_O_2_ and neutrophil killing as well as virulence. We further find that the downregulation of the oxidative stress response genes is due to defective expression of the stationary-phase sigma factor RpoS. We demonstrate an autoregulatory mechanism of RpoS and find that *ybeY* mutation increases the level of a small RNA, ReaL, which directly represses the translation of *rpoS* through the 5′ UTR of its mRNA and subsequently reduces the expression of the oxidative stress response genes. *In vitro* assays demonstrate direct degradation of ReaL by YbeY. Deletion of *reaL* or overexpression of *rpoS* in the *ybeY* mutant restores the bacterial tolerance to oxidative stress and the virulence. We also demonstrate that YbeZ binds to YbeY and is involved in the 16S rRNA processing and regulation of *reaL* and *rpoS* as well as the bacterial virulence. Overall, our results reveal pleiotropic roles of YbeY and the YbeY-mediated regulation of *rpoS* through ReaL.

## Abstract (precis)

The increasing bacterial antibiotic resistance imposes a severe threat to human health. For the development of effective treatment and prevention strategies, it is critical to understand the mechanisms employed by bacteria to grow in the human body. Posttranscriptional regulation plays an important role in bacterial adaptation to environmental changes. RNases and small RNAs are key players in this regulation. In this study, we demonstrate critical roles of the RNase YbeY in the virulence of the pathogenic bacterium Pseudomonas aeruginosa. We further identify the small RNA ReaL as the direct target of YbeY and elucidate the YbeY-regulated pathway on the expression of bacterial virulence factors. Our results shed light on the complex regulatory network of P. aeruginosa and indicate that inference with the YbeY-mediated regulatory pathway might be a valid strategy for the development of a novel treatment strategy.

## INTRODUCTION

Successful colonization of the host by a pathogenic bacterium depends on efficient orchestration of global gene expression to quickly adapt to the host *in vivo* environment and evade the immune clearance (1). In response to environmental changes, bacteria control gene expression through transcriptional, posttranscriptional, and posttranslational mechanisms (2). Compared to transcriptional regulation that involves RNA synthesis, the posttranscriptional processing provides a way of regulation that saves time and energy as the mRNA translation and stability are regulated by RNases and small RNAs (sRNAs) (3–5). By binding to target mRNAs, sRNAs affect ribosome accessibility or RNase-mediated cleavage. Meanwhile, the processing and stabilities of sRNAs are under the control of RNases (6–8). Therefore, identification of the target sRNAs and mRNAs of RNases is essential for the elucidation of the RNase-mediated regulatory pathways.

Pseudomonas aeruginosa is an opportunistic Gram-negative pathogen that causes acute and chronic infections in human (9). Upon infection, phagocytes play an essential role in the host defense against pathogenic bacteria. One of the major bacterial killing mechanisms of phagocytes is the generation and release of reactive oxygen species (ROS) (10, 11). P. aeruginosa harbors a variety of antioxidant enzymes, including catalases KatA and KatB and alkyl hydroperoxide reductase AphB, AhpC, and AhpF (12, 13). KatA is a constitutively expressed catalase that plays a major role in the bacterial defense against oxidative stresses and virulence (14–16). Both KatA and KatB are regulated at the transcriptional level by a variety of factors, including OxyR and the stationary-phase sigma factor RpoS (17, 18). Mutation of *rpoS* reduces the expression of these catalases, leading to increased susceptibility to oxidative stresses and attenuation of virulence in animal models (17, 19, 20).

RNases have been shown to play important roles in the regulation of virulence determinants in P. aeruginosa. Previously, we found that the polynucleotide phosphorylase (PNPase) controls the expression of the type III (T3SS) and type VI secretion systems and pyocin synthesis genes (21, 22). We also found that the PNPase degrades sRNA P27, which directly controls the translation of the quorum sensing signal synthase RhlI (23). In P. aeruginosa, PNPase interacts with RNase E and RNA helicase DeaD to form an RNA degradosome that plays an important role in RNA processing (24). Both RNase E and DeaD are required for the expression of the T3SS genes (25–27).

YbeY is a highly conserved bacterial RNase that is involved in the maturation of 16S rRNA, ribosome quality control, regulation of sRNA, and stress responses (28–33). In pathogenic bacteria, such as enterohemorrhagic Escherichia coli (EHEC), Vibrio cholerae, and Yersinia enterocolitica, YbeY has been shown to play important roles in bacterial virulence (29, 34, 35). However, the mechanisms by which YbeY affects bacterial virulence and stress response remain unclear. Among bacterial species, including P. aeruginosa, E. coli, and Staphylococcus aureus, the *ybeY* gene is colocalized with a *ybeZ* gene in the same operon (36–38). In E. coli, YbeY has been found to interact with YbeZ (37), indicating a functional connection between the two proteins. YbeZ contains an ATP binding and a nucleoside triphosphate hydrolase domain; however, its exact function remains unknown.

In this study, we demonstrate that the P. aeruginosa endoribonuclease YbeY is involved in the 16S rRNA maturation, ribosome assembly, and pathogenesis. We further identify the sRNA ReaL as the target of YebY and elucidate a YbeY-mediated regulatory pathway that controls the expression of *rpoS* and oxidative stress response genes. In addition, we elucidate a posttranscriptional regulatory mechanism of RpoS as well as a functional connection between YbeZ and YbeY.

## RESULTS

### YbeY of P. aeruginosa is essential for the 16S rRNA maturation and ribosome assembly.

**FIG 1:**
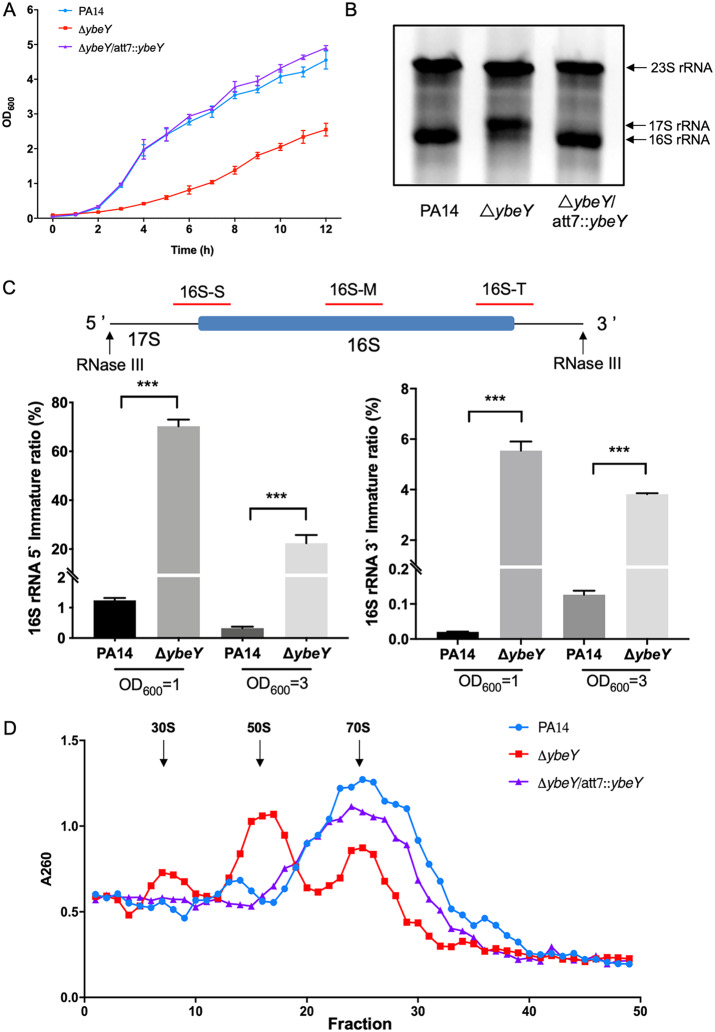
YbeY influences the growth rate, 16S rRNA maturation, and ribosome assembly in P. aeruginosa. (A) Bacterial growth rates. Same numbers of cells of the indicated strains were inoculated in LB. The OD_600_ was monitored every hour for 12 h. (B) Bacterial cells were cultured in LB to an OD_600_ of 1, followed by RNA isolation. The 23S and 16S rRNA and the 16S rRNA precursor (17S) were separated by electrophoresis. (C) Top: Schematic diagram of the 16S rRNA precursor. Black arrows indicate the processing sites of RNase III. The red bars represent the regions amplified by real-time PCR. 16S-S, 16S-M, and 16S-T represent the 5′ starting site, middle, and 3′ termination site of the 16S rRNA, respectively. The bacteria were grown in LB to the OD_600_ of 1 or 3. The bacterial total RNA was isolated, and the 5′ and 3′ immature ratios of the 16S rRNA were determined by real-time PCR. ***, *P* < 0.001 by Student’s *t* test. (D) Bacteria were grown to an OD_600_ of 1. The ribosome particles were subjected to sucrose gradient separation and quantified by UV absorbance at 260 nm.

In P. aeruginosa, gene PA3982 (PA14_12310 in the PA14 genome) encodes an YbeY homolog. To examine the function of YbeY in P. aeruginosa, we constructed a *ybeY* mutant in wild-type PA14. Deletion of the *ybeY* gene reduced the bacterial growth rate (Fig. 1A). We then examined its role in the maturation of the 16S rRNA. The Δ*ybeY* mutant showed an increased proportion of the 16S rRNA precursor, which was restored to the wild-type level by complementation with a *ybeY* gene (Fig. 1B). To explore how YbeY influences the maturation of 16S rRNA, we designed three pairs of real-time PCR (RT-PCR) primers targeting the middle, 5′ starting site, and 3′ termination site of the 16S rRNA, representing the total, 5′ immature, and 3′ immature 16S rRNA, respectively. In both the logarithmic and stationary growth phases, the proportion of immature 16S rRNA in the Δ*ybeY* mutant was higher than that in wild-type PA14, with the 5′ immature ratio much higher than the 3′ immature ratio (Fig. 1C). We then examined the role of YbeY in ribosome assembly. Deletion of the *ybeY* gene reduced the proportion of assembled 70S ribosome while increasing the proportion of unassembled 30S and 50S ribosome components (Fig. 1D).

In previous studies, the *ybeY* mutant was not identified from Tn mutant libraries, indicating that *ybeY* might be an essential gene in P. aeruginosa (29, 39). To examine whether *ybeY* is essential for P. aeruginosa, we deleted the gene in another two wild-type strains, PAO1 and PAK. Similar to the PA14 Δ*ybeY* mutant, the Δ*ybeY* mutants of PAO1 and PAK displayed reduced growth rate and increased proportion of 16S rRNA precursor (see Fig. S1 in the supplemental material). These results suggest that YbeY plays important roles in the rRNA processing and growth of P. aeruginosa.

10.1128/mBio.00659-20.1 FIG S1 YbeY influences the growth rates and 16S rRNA maturation in PAO1 and PAK. (A and B) Bacterial growth rates. Same numbers of cells of the indicated strains were inoculated in LB. The OD_600_ was monitored every hour for 12 h. (C and D) The bacterial total RNA was isolated, and the 5′ and 3′ immature ratios of the 16S rRNA were determined by real-time PCR. ***, *P* < 0.001 by Student’s *t* test. Download FIG S1, PDF file, 0.2 MB. Copyright © 2020 Xia et al. 2020 Xia et al. This content is distributed under the terms of the Creative Commons Attribution 4.0 International license.

To confirm that the P. aeruginosa *ybeY* (PA3982) gene is a true ortholog of the *ybeY* gene in E. coli, we complemented the PA14 Δ*ybeY* mutant with an E. coli *ybeY* gene, which restored the bacterial growth rate and maturation of the 16S rRNA (Fig. S2A, B, and C). Consistent with previous studies, the E. coli *ybeY* mutant was more susceptible to heat shock and oxidative stresses (such as H_2_O_2_) (29). Complementation of the E. coli *ybeY* mutant with the *ybeY* gene from PA14 restored the bacterial resistance to heat shock and H_2_O_2_ (Fig. S2D and E).

10.1128/mBio.00659-20.2 FIG S2 Functional similarities between the P. aeruginosa and E. coli YbeY. (A) Same numbers of cells of the indicated strains were inoculated in LB. The OD_600_ was monitored every hour for 12 h. (B and C) The bacteria were grown to an OD_600_ of 1.0, followed by total RNA isolation. The 5′ and 3′ immature ratios of the 16S rRNA were determined by real-time PCR. (D) The bacteria were grown to an OD_600_ of 1.0 at 37°C and then incubated at 45°C for 2 h. The live bacterial numbers were determined by serial dilution and plating. (E) The bacteria were cultured to an OD_600_ of 1 and then washed three times with PBS and resuspended in PBS. The bacteria were treated with 10, 20, or 50 mM H_2_O_2_ at 37°C for 30 min. The live bacterial numbers were determined by serial dilution and plating. The survival rate was calculated by comparing the live bacterial number after the treatment with that before the treatment. *, *P* < 0.05; **, *P* < 0.01; ***, *P* < 0.001 by Student’s *t* test. Download FIG S2, PDF file, 0.2 MB. Copyright © 2020 Xia et al. 2020 Xia et al. This content is distributed under the terms of the Creative Commons Attribution 4.0 International license.

**FIG 2:**
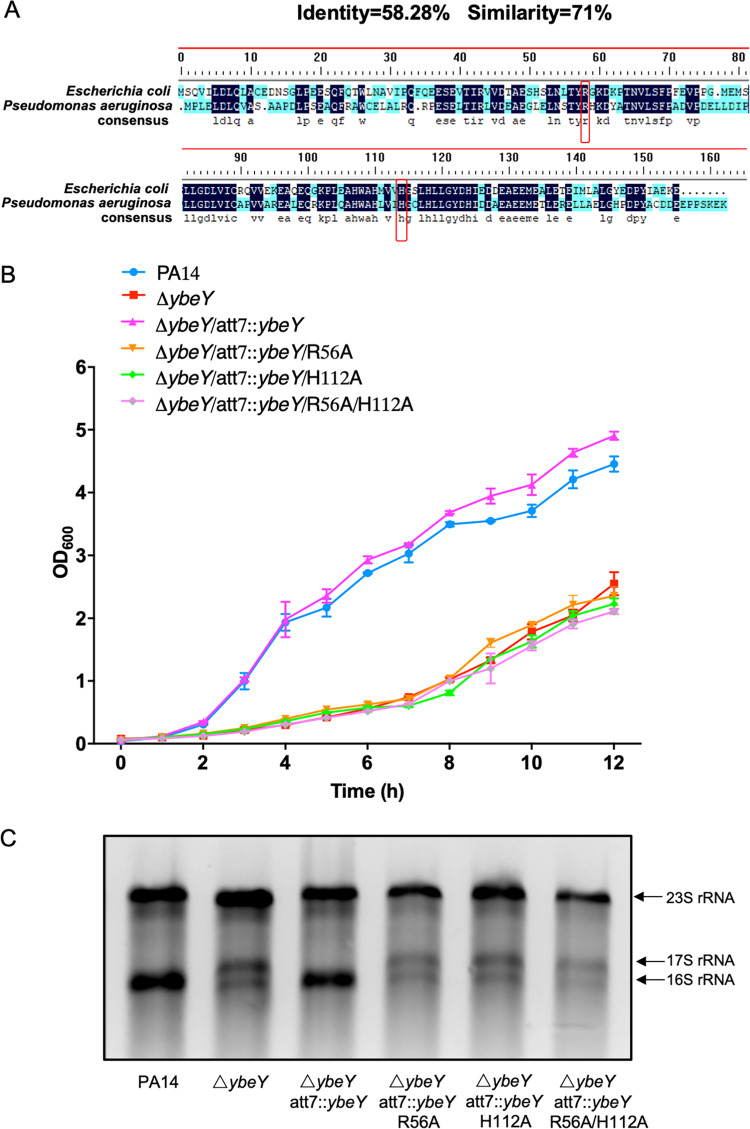
The amino acid residues R56 and H112 are critical for the function of YbeY. (A) Sequence alignment of P. aeruginosa and E. coli YbeY. Identical amino acids are indicated by dark blue; the similar amino acids are indicated by lighter blue. The conserved R56 and H112 are indicated by red boxes. (B) Bacterial growth rates in LB. Same numbers of cells of the indicated strains were inoculated in LB. The OD_600_ was monitored every hour for 12 h. (C) The bacteria were cultured in LB to an OD_600_ of 1, followed by RNA isolation. The 23S and 16S rRNA and the 16S rRNA precursor (17S) were separated by electrophoresis.

Previous studies in E. coli and Bacillus subtilis revealed that the amino acid residues R56 and H112 are critical for the function of YbeY (28, 40, 41). To determine the importance of these conserved amino acid residues in the P. aeruginosa YbeY, we replaced the R56 or H112 with alanine (A). Neither of the alleles restored the maturation of the 16S RNA and the growth rate of the Δ*ybeY* mutant (Fig. 2B and C), indicating the essentialities of these residues in the function of the P. aeruginosa YbeY.

### YbeZ binds to YbeY and contributes to the processing of the 16S rRNA.

**FIG 3:**
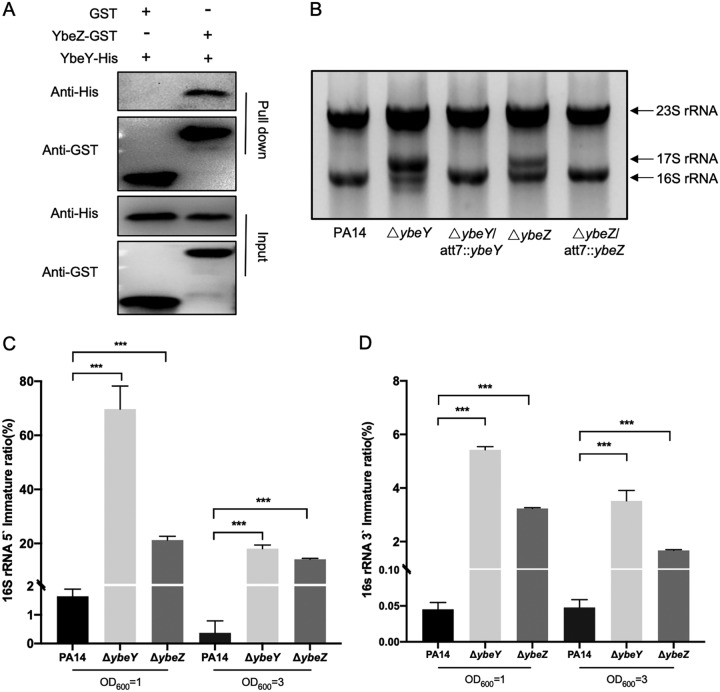
YbeZ binds to YbeY and contributes to the processing of the 16S rRNA. (A) Examination of the interaction between YbeY and YbeZ by a pulldown assay. Cell lysates containing the YbeY-His were incubated with the resin bound with GST or YbeZ-GST for 2 h. The beads were washed three times with the cell lysis buffer. The bound proteins were eluted with GSH and subjected to Western blotting. (B) Bacterial cells were cultured in LB to an OD_600_ of 1, followed by RNA isolation. The 23S and 16S rRNA and the 16S rRNA precursor (17S) were separated by electrophoresis. (C and D) The 5′ immature (C) and 3′ immature (D) ratio of 16S rRNA was determined by real-time PCR. **, *P* < 0.01; ***, *P* < 0.001 by Student’s *t* test.

In the P. aeruginosa genome, *ybeY* (PA3982) is predicted to be in the same operon with *ybeZ* (PA3981) and *ybeX* (PA3983). RT-PCR results confirmed the cotranscription of these three genes (Fig. S3). A previous study in E. coli revealed the interaction between YbeY and YbeZ (37), suggesting that YbeZ might function as a partner of YbeY. To test whether YbeY and YbeZ are functionally connected in P. aeruginosa, we performed a pulldown assay. Our results revealed active interaction between YbeY and YbeZ (Fig. 3A). Next, we examined the biological function of YbeZ. Mutation of *ybeZ* also reduced the bacterial growth rate (Fig. S4A) and maturation of the 16S rRNA, but to a lesser extent compared to the *ybeY* mutation (Fig. 3B to D). For example, at the OD_600_ of 1.0, the 16S rRNA 5′ and 3′ immature ratios of the Δ*ybeZ* mutant were approximately 13- and 60-fold higher than those in the wild-type PA14, respectively, whereas the corresponding differences between the Δ*ybeY* mutant and the wild-type PA14 were approximately 43- and 100-fold (Fig. 3C and D). However, mutation of *ybeZ* did not affect the assembly of the ribosome (Fig. S4B). These results indicate that YbeY and YbeZ might form a complex that processes the 16S rRNA precursor, with YbeY playing a major role. Accordingly, we focused our following studies on the functions of YbeY.

10.1128/mBio.00659-20.3 FIG S3 PA3981, PA3982, and PA3983 are in one operon. (A) Locations of the primers. (B) PCR products from indicated primers and templates. Download FIG S3, PDF file, 0.3 MB. Copyright © 2020 Xia et al. 2020 Xia et al. This content is distributed under the terms of the Creative Commons Attribution 4.0 International license.

10.1128/mBio.00659-20.4 FIG S4 Roles of YbeZ in the bacterial growth and ribosome assembly. (A) Growth rate of the indicated strains in LB medium. Overnight cultures of indicated strains were 1:100 diluted into fresh LB. The bacterial growth was monitored by measuring OD_600_ every hour for 12 h. (B) Ribosome profiles from PA14 and the Δ*ybeZ* and Δ*ybeZ*/att7::*ybeZ* strains. The bacteria were grown to an OD_600_ of 1. The ribosome particles were subjected to sucrose gradient separation and quantified by UV absorbance at 260 nm. Download FIG S4, PDF file, 0.1 MB. Copyright © 2020 Xia et al. 2020 Xia et al. This content is distributed under the terms of the Creative Commons Attribution 4.0 International license.

### YbeY is required for the virulence of P. aeruginosa in a murine acute pneumonia model.

**FIG 4:**
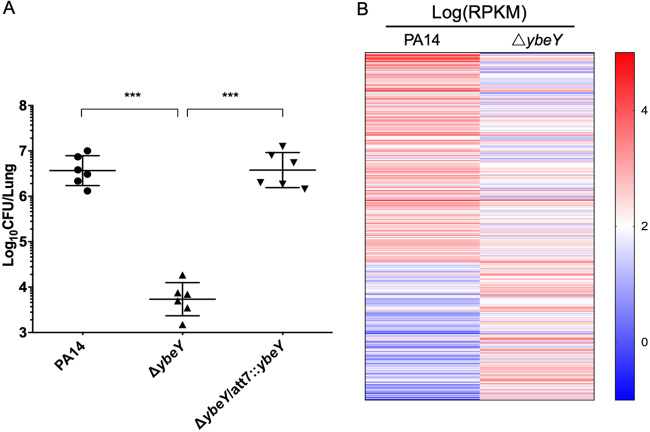
YbeY is required for the virulence of P. aeruginosa in the acute pneumonia model. (A) Mice were infected intranasally with the indicated strains. At 12 h postinfection, lungs from the infected mice were isolated. The bacterial loads were determined by serial dilution and plating. ***, *P* < 0.001 by Student’s *t* test. (B) RNA-seq results. Values reported as log(RPKM) of the genes with more than 2-fold difference in expression between the Δ*ybeY* mutant and the wild-type PA14.

To examine the role of YbeY in the virulence of P. aeruginosa, we utilized a murine acute pneumonia model. Mutation of *ybeY* reduced the bacterial load by approximately 600-fold, which was restored by the complementation with a wild-type *ybeY* gene (Fig. 4A).

**FIG 5:**
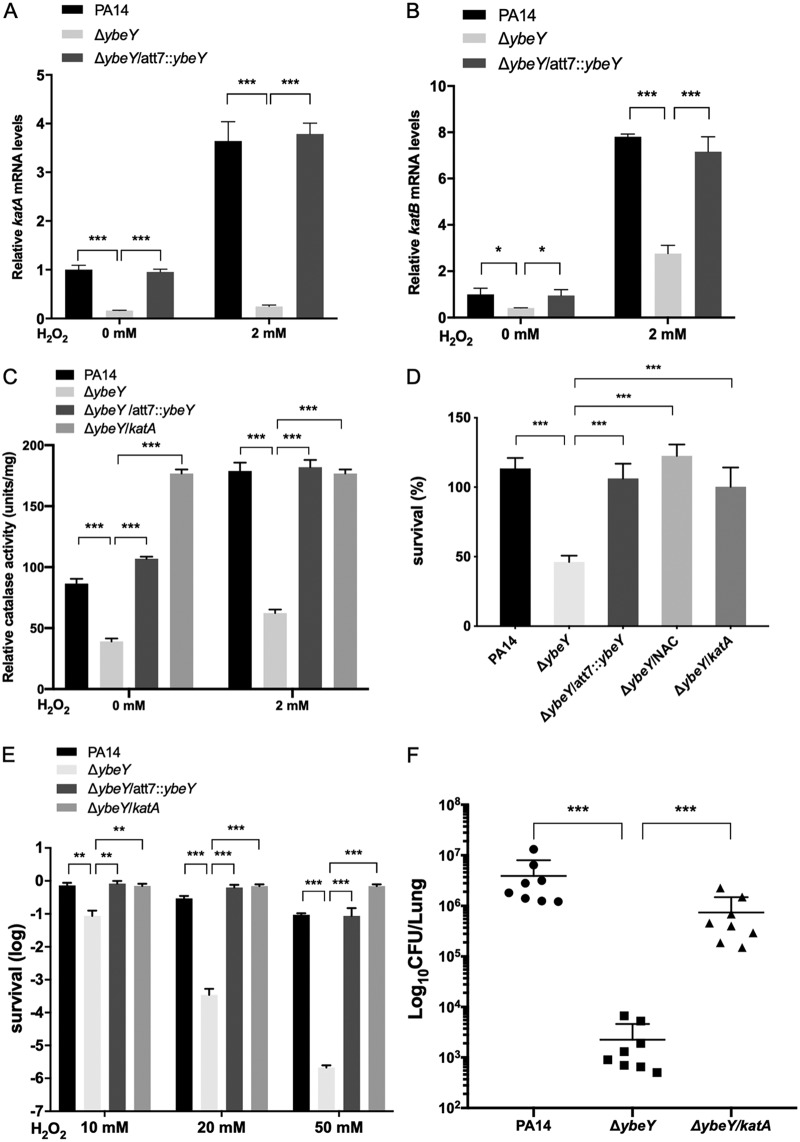
Mutation of *ybeY* reduces the bacterial response to oxidative stress and virulence. Wild-type PA14, Δ*ybeY* mutant, and the complemented strain were grown in LB to an OD_600_ of 1 and then incubated with or without 2 mM H_2_O_2_ for 30 min. (A and B) The relative mRNA levels of *katA* (A) and *katB* (B) were determined by real-time PCR. Results represent means ± SD. (C) The indicated strains were grown in LB to an OD_600_ of 1 and then incubated with or without 2 mM H_2_O_2_ for 30 min. The bacteria were collected by centrifugation, and the cells were broken by sonication. The total intracellular catalase activity was measured using a catalase assay kit. (D) The indicated strains were incubated with dHL60 cells at an MOI of 5 in HBSS or HBSS with 80 mM *N*-acetylcysteine for 3 h. The bacterial survival rates were determined by plating. (E) The indicated strains were grown in LB to an OD_600_ of 1. The cells were washed three times with PBS and then incubated with 10, 20, or 50 mM H_2_O_2_ for 30 min. The bacteria were collected by centrifugation and resuspended with fresh LB, and the survival rates were determined by plating. (F) Mice were infected intranasally with the indicated strains. At 12 h postinfection, lungs from the infected mice were isolated. The bacterial loads were determined by serial dilution and plating. *, *P* < 0.05; **, *P* < 0.01; ***, *P* < 0.001 by Student’s *t* test.

To understand the mechanism of YbeY-mediated regulation on the bacterial virulence, we performed transcriptome analyses. Expression of 313 genes was altered by the mutation of *ybeY* (Fig. 4B; Table S2). Of note, genes involved in the oxidative stress response were downregulated in the Δ*ybeY* mutant, including the catalase genes *katA* and *katB*, the alkyl hydroperoxide reductase gene *aphC*, and the superoxide dismutase gene *sodB* (Table 1). The catalases play critical roles during P. aeruginosa infection. KatA is a constitutive and housekeeping catalase produced by P. aeruginosa and plays a critical role in the bacterial tolerance to oxidative stresses and virulence (42, 43), whereas the expression of *katB* is induced by oxidative stresses (15). Mutation of *yebY* reduced the mRNA levels of *katA* and *katB* in the presence and absence of H_2_O_2_, which were restored by complementation with the *yebY* gene (Fig. 5A and B). Consistent with the gene expression pattern, the total catalase activity of the Δ*ybeY* mutant was lower than that of the wild-type PA14, which was restored by overexpression of *katA* (Fig. 5C).

**TABLE 1:** mRNA levels of oxidative response genes in the Δ*ybeY* mutant compared to those in wild-type PA14

Gene name	Product	Fold change Δ*ybeY*/PA14	*P* value
*katA*	Catalase	0.03	3.31E−07
*katB*	Catalase	0.11	9.11E−12
*ahpC*	Alkyl hydroperoxide reductase	0.11	4.88E−07
*sodB*	Superoxide dismutase	0.28	7.76E−04
*rpoS*	RNA polymerase sigma factor RpoS	0.34	9.11E−12

In the acute pneumonia model, the neutrophil plays an important role in the host clearance of the invading P. aeruginosa (12). Generation of ROS is one of the major bacterium-killing mechanisms by the neutrophil (11, 12). The downregulation of the oxidative response genes might render the Δ*ybeY* mutant more susceptible to the neutrophil-mediated killing. Indeed, after incubation with neutrophils differentiated from HL60 cells (dHL60), the survival rate of the Δ*ybeY* mutant was significantly lower than that of the wild-type strain (Fig. 5D). Supplementation of the ROS scavenger molecule *N*-acetylcysteine (NAC) or overexpression of *katA* restored the bacterial survival rate (Fig. 5D). In addition, the Δ*ybeY* mutant was more susceptible to H_2_O_2_ treatment and overexpression of the *katA* gene restored the bacterial survival rate (Fig. 5E). Furthermore, overexpression of *katA* in the Δ*ybeY* mutant restored the bacterial load in the acute pneumonia model (Fig. 5F) without affecting the bacterial growth rate in LB (Fig. S5). These results indicate that the defective bacterial response to ROS contributes to the attenuated virulence of the Δ*ybeY* mutant.

10.1128/mBio.00659-20.5 FIG S5 Growth rate of indicated strains in LB medium. Overnight cultures of the indicated strains were 1:100 diluted into fresh LB. The bacterial growth was monitored by measuring OD_600_ every hour for 12 h. Download FIG S5, PDF file, 0.1 MB. Copyright © 2020 Xia et al. 2020 Xia et al. This content is distributed under the terms of the Creative Commons Attribution 4.0 International license.

### YbeY controls the oxidative stress response genes through RpoS.

In P. aeruginosa, the transcriptional regulator OxyR controls the expression of the catalase and the alkyl hydroperoxide reductase genes in response to oxidative stresses (44). In addition, the ATP-dependent helicase RecG facilitates the binding of OxyR to the promoters of target genes (45, 46). In the Δ*ybeY* mutant, the expression levels of *oxyR*, *recG*, and *ahpB* (alkyl hydroperoxide reductase) were similar to those in the wild-type PA14 in the presence and absence of H_2_O_2_ (Fig. S6). The *aphC* mRNA level in the Δ*ybeY* mutant was lower in the absence of H_2_O_2_ but increased to a similar level as that in PA14 in the presence of H_2_O_2_. These results indicate that YbeY might not control the expression of *katA*/*B* through OxyR and RecG.

10.1128/mBio.00659-20.6 FIG S6 Expression of oxidative stresses response-related genes. Wild-type PA14, the Δ*ybeY* mutant, and the complemented strain were grown in LB to an OD_600_ of 1 and then incubated with or without 2 mM H_2_O_2_ for 30 min. The relative mRNA levels were determined by real-time PCR. Results represent means ± SD. Download FIG S6, PDF file, 0.1 MB. Copyright © 2020 Xia et al. 2020 Xia et al. This content is distributed under the terms of the Creative Commons Attribution 4.0 International license.

**FIG 6:**
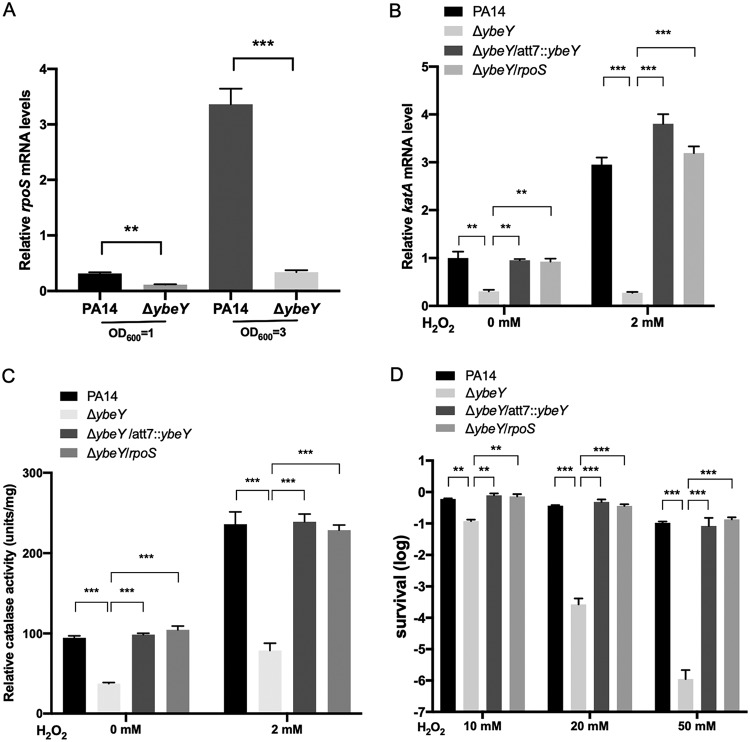
YbeY controls the bacterial response to oxidative stresses through *rpoS*. (A) PA14 and the Δ*ybeY* mutant were cultured in LB to an OD_600_ of 1 or 3. The relative mRNA levels of *rpoS* were determined by real-time PCR. Results represent means ± SD. (B and C) The indicated strains were grown in LB to an OD_600_ of 1 and then incubated with or without 2 mM H_2_O_2_ for 30 min. (B) The relative mRNA levels of *katA* were determined by real-time PCR. Results represent means ± SD. (C) The bacteria were collected by centrifugation, and the cells were broken by sonication. The total intracellular catalase activity was measured using a catalase assay kit. (D) The indicated strains were grown in LB to an OD_600_ of 1, and the cells were washed three times with PBS and then incubated with 10, 20, or 50 mM H_2_O_2_ for 30 min. The bacteria were collected by centrifugation and resuspended with fresh LB, and the survival rates were determined by plating. ns, not significant; *, *P* < 0.05; **, *P* < 0.01; ***, *P* < 0.001 by Student’s *t* test.

Besides OxyR and RecG, the alternative sigma factor RpoS had been demonstrated to affect the expression of *katA*/*B* and the bacterial tolerance to H_2_O_2_ (18–20). Our RNA-seq result revealed downregulation of the *rpoS* gene in the Δ*ybeY* mutant (Table 1), which was confirmed by real-time PCR (Fig. 6A). Overexpression of *rpoS* in the Δ*ybeY* mutant restored the expression of *katA* (Fig. 6B) and the total catalase activity in the presence and absence of H_2_O_2_ (Fig. 6C) as well as the bacterial tolerance to H_2_O_2_ (Fig. 6D). In addition, mutation of *rpoS* did not affect the expression of *ybeY* at either the logarithmic or stationary growth phase (Fig. S7). In combination, these results demonstrate that YbeY controls the expression of *katA* through RpoS.

10.1128/mBio.00659-20.7 FIG S7 Expression of *ybeY*. Wild-type PA14 and the *rpoS*::Tn mutant were grown in LB to an OD_600_ of 1 or 3. The relative mRNA levels of *ybeY* were determined by real-time PCR. Results represent means ± SD. ns, not significant by Student’s *t* test. Download FIG S7, PDF file, 0.1 MB. Copyright © 2020 Xia et al. 2020 Xia et al. This content is distributed under the terms of the Creative Commons Attribution 4.0 International license.

### YbeY controls the expression of *rpoS* at the posttranscriptional level.

**FIG 7:**
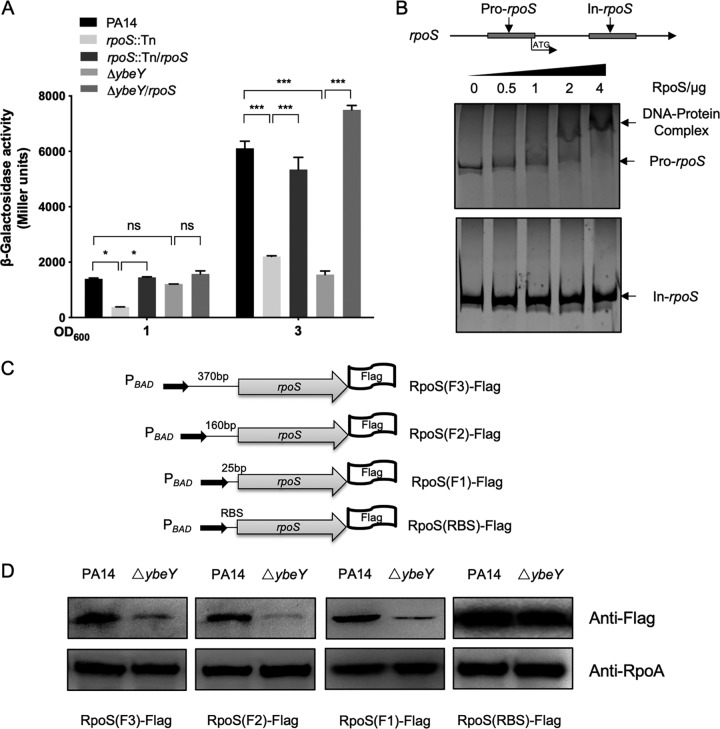
YbeY affects the translation of *rpoS* through a 25-nucleotide sequence in the 5′ UTR. (A) The indicated strains containing P*_rpoS_*-*lacZ* transcriptional fusion were cultured in LB to an OD_600_ of 1 or 3. The bacteria were collected and subjected to the β-galactosidase activity assay. ns, not significant; *, *P* < 0.05; ***, *P* < 0.001 by Student’s *t* test. (B) The 6×His-tagged RpoS protein was expressed in E. coli and purified through Ni-NTA affinity chromatography. Pro-*rpoS* and In-*rpoS* represent the fragments of the *rpoS* promoter and coding regions, respectively. The location of the fragments was indicated by arrows. One hundred nanograms purified DNA fragment was incubated with the indicated amounts of RpoS protein for 30 min, followed by electrophoresis in a nondenatured polyacrylamide gel. The arrow indicates the DNA-protein complex. (C) Diagrams of C-terminal Flag-tagged *rpoS* fusions (*rpoS*-Flag) driven by an exogenous arabinose-inducible promoter (P*_BAD_*) with various lengths of the *rpoS* 5′ UTR. RBS, ribosome binding sequence from the vector pET28a. (D) PA14 and its Δ*ybeY* mutant strain containing the individual RpoS translation fusions were cultured in LB to an OD_600_ of 1 and then incubated with 0.2% l-arabinose for 60 min. The bacteria were collected for Western blot analysis.

We then explored the mechanism of YbeY-mediated regulation of *rpoS*. The transcription of *rpoS* was examined using a transcriptional fusion between the *rpoS* promoter and a promoterless *lacZ* reporter gene (P*_rpoS_*-*lacZ*). β-Galactosidase assay revealed a reduced *rpoS* promoter activity in the Δ*ybeY* mutant (Fig. 7A). A previous ChIP-seq analysis indicated a possible binding of RpoS to its own promoter, suggesting an autoregulation (47). To verify whether RpoS controls its own expression, we utilized an *rpoS*::Tn mutant from the PA14 transposon insertion mutant library (48). Mutation in *rpoS* reduced the LacZ expression from P*_rpoS_*-*lacZ*, which was restored by overexpression of an *rpoS* gene driven by an exogenous *tac* promoter (Fig. 7A). An EMSA demonstrated that RpoS indeed bound to its own promoter but not the coding sequence (Fig. 7B). Collectively, these results suggest an autoregulation of *rpoS*.

To elucidate the mechanism of YebY-mediated regulation of *rpoS*, we overexpressed the *rpoS* gene by a constitutively active *tac* promoter in the Δ*ybeY* mutant, which restored the expression of the P*_rpoS_*-*lacZ* (Fig. 7A). These results raise the possibility that the lower *rpoS* promoter activity in the Δ*ybeY* mutant might be due to a deficiency in the translation of the *rpoS* mRNA. To examine the translation of *rpoS*, we constructed a series of C-terminal Flag-tagged *rpoS* fusions (*rpoS*-Flag) driven by an exogenous arabinose-inducible promoter (P*_BAD_*) with various lengths of the *rpoS* 5′ UTR, resulting in *rpoS*(F1)-FLAG, *rpoS*(F2)-FLAG, and *rpoS*(F3)-FLAG (Fig. 7C). The translation of *rpoS* was reduced in the Δ*ybeY* mutant even when the 5′ UTR was truncated to 25 nucleotides upstream of the start codon. However, when the 25-nucleotide sequence was replaced by an exogenous ribosome binding sequence from vector plasmid pET28a, the RpoS-FLAG protein levels were similar between wild-type PA14 and the Δ*ybeY* mutant (Fig. 7C and D). In combination, these results demonstrate that YbeY affects the translation of *rpoS.*

### YbeY controls the *rpoS* translation through sRNA ReaL.

**FIG 8:**
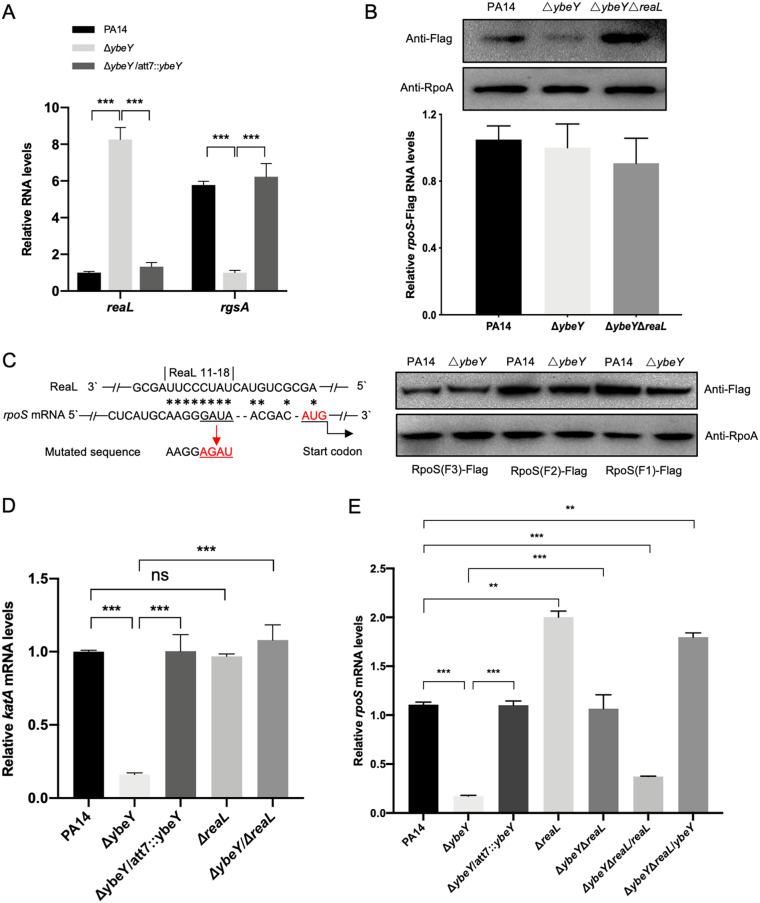
YbeY controls the translation of RpoS through ReaL. (A) Wild-type PA14, the Δ*ybeY* mutant, and the complemented strain were grown in LB to an OD_600_ of 1. The relative levels of the sRNAs *reaL* and *rgsA* were determined by real-time PCR. Results represent means ± SD. ***, *P* < 0.001 by Student’s *t* test. (B) PA14, the Δ*ybeY* mutant, and the Δ*ybeY* Δ*reaL* mutant containing the RpoS(F1)-FLAG translational fusion were cultured in LB to an OD_600_ of 1 and then incubated with 0.2% l-arabinose for 60 min. The bacteria were collected for Western blot analysis. The relative mRNA levels of the *rpoS*-Flag were determined by real-time PCR using the primers matching to the Flag tag RNA. The results represent means ± SD. ns, not significant. (C) The potential base-pairing between ReaL and the 5′ UTR is indicated by asterisks. The mutated nucleotides are underlined. PA14 and the Δ*ybeY* mutant containing the indicated *rpoS*-Flag fusions were cultured in LB to an OD_600_ of 1 and then incubated with 0.2% l-arabinose for 60 min. The bacteria were collected for Western blot analysis. (D and E) The indicated strains were grown in LB to an OD_600_ of 1. The relative mRNA levels of *katA* (D) and *rpoS* (E) were determined by real-time PCR. The results represent means ± SD. **, *P* < 0.01 by Student’s *t* test; ***, *P* < 0.001 by Student’s *t* test.

In P. aeruginosa, two sRNAs, namely, RgsA and ReaL, repress the *rpoS* translation by targeting to the −25 to +27 (relative to the start codon) and the Shine-Dalgarno (SD) regions (−13 to −6) of the *rpoS* mRNA, respectively (3, 49, 50). In the Δ*ybeY* mutant, *rgsA* was downregulated whereas *reaL* was upregulated (Fig. 8A), indicating a possible involvement of ReaL in the repression of the *rpoS* translation. Indeed, deletion of *reaL* in the Δ*ybeY* mutant restored the translation of the P*_BAD_*-driven *rpoS*-FLAG while the mRNA levels of the *rpoS*-FLAG were similar among the strains (Fig. 8B).

To further verify that the repression of *rpoS* is due to the upregulation of *reaL*, we mutated the predicted binding site of ReaL (3, 49, 50) in the *rpoS*(F1)-FLAG, *rpoS*(F2)-FLAG, and *rpoS*(F3)-FLAG (Fig. 8C), which resulted in similar expression levels of the RpoS-FLAG in wild-type PA14 and the Δ*ybeY* mutant (Fig. 8C). Meanwhile, deletion of *reaL* in the Δ*ybeY* mutant background restored the mRNA levels of *katA* and *rpoS* (Fig. 8D and E). Overexpression of *reaL* in the double-knockout mutant reduced the *rpoS* mRNA level, whereas overexpression of *ybeY* resulted in a higher *rpoS* mRNA level than that in the wild-type PA14 (Fig. 8E). These results indicate that the high level of ReaL in the Δ*ybeY* mutant contributes to the downregulation of *rpoS* and that YbeY might positively regulate *rpoS* through other mechanisms.

### YbeY directly degrades ReaL.

**FIG 9:**
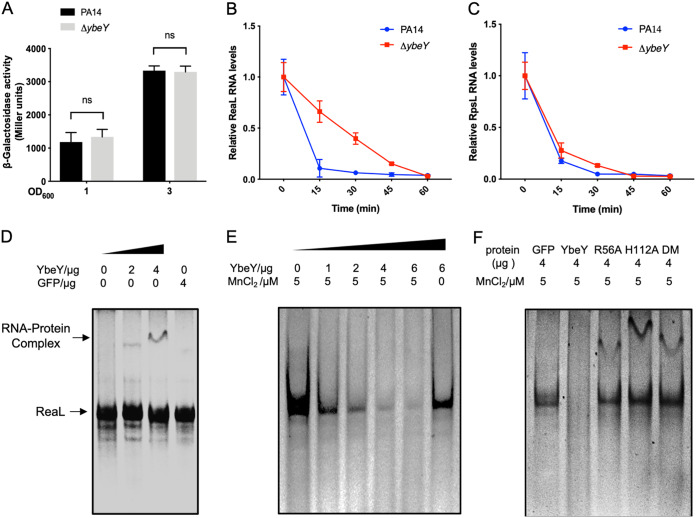
YbeY directly degrades ReaL. (A) PA14 and the Δ*ybeY* mutant containing the P*_reaL_*-*lacZ* transcriptional fusion were cultured in LB to an OD_600_ of 1 or 3. The bacteria were collected and subjected to the β-galactosidase activity assay. ns, not significant. (B and C) Degradation of ReaL (B) and the *rpsL* mRNA (C) in wild-type PA14 and the Δ*ybeY* mutant. Bacterial cells were treated with rifampin to stop the transcription. At indicated time points, the bacteria were collected and mixed with equal numbers of *gfp*-expressing E. coli cells. Total RNA was isolated, and the relative RNA levels were determined by real-time PCR. The *gfp* mRNA in each sample was used as the internal control for normalization. (D) The 6×His-tagged YbeY protein was expressed in E. coli and purified through Ni-NTA affinity chromatography. One hundred nanograms purified RNA transcript was incubated with the indicated amounts of the YbeY protein with an RNase inhibitor for 30 min on ice, followed by electrophoresis in a nondenatured polyacrylamide gel. The arrow indicates the RNA-protein complex. (E and F) RNA degradation by wild-type (E) and the mutated (F) YbeY. Purified GFP, wild-type YbeY, and those with indicated mutations were incubated with 100 ng purified ReaL at 37°C for 30 min with indicated concentrations of MnCl_2_. Then the samples were analyzed by electrophoresis in a nondenatured polyacrylamide gel. The RNA bands were visualized by staining with Gel-red (Biotium).

To explore the mechanism of the upregulation of ReaL, we constructed a transcriptional fusion of the *reaL* promoter with a promoterless *lacZ* gene (P*_reaL_*-*lacZ*). The LacZ levels were similar in wild-type PA14 and the Δ*ybeY* mutant (Fig. 9A), indicating a similar transcriptional level of the sRNA. We then examined the stability of ReaL. Following blockage of the RNA synthesis by rifampin, the ReaL level dropped quickly in wild-type PA14, whereas a slower reduction was observed in the Δ*ybeY* mutant (Fig. 9B). Meanwhile, the mRNA level of RpsL (a ribosomal subunit) dropped at a similar rate in the two strains (Fig. 9C). These results suggest that the higher level of ReaL in the Δ*ybeY* mutant is likely due to its increased stability.

To address whether ReaL is a direct target of YbeY, we tested whether ReaL can interact with YbeY by EMSA in the presence of an RNase inhibitor. As shown in Fig. 9D, YbeY retarded the migration of ReaL, indicating a direct interaction. Next, we investigated whether YbeY can degrade ReaL directly. As shown in Fig. 9E and F, the presence of Mn^2+^ significantly increased the degradation of the ReaL RNA by YbeY, which is consistent with previous reports that YbeY is a metal-dependent endoribonuclease (1, 6, 10). Furthermore, mutation of the conserved residues R56 and H112 or both of them in YbeY reduced the degradation efficacy (Fig. 9E and F).

### Downregulation of *rpoS* by ReaL plays a major role in the attenuated virulence of the Δ*ybeY* mutant.

**FIG 10:**
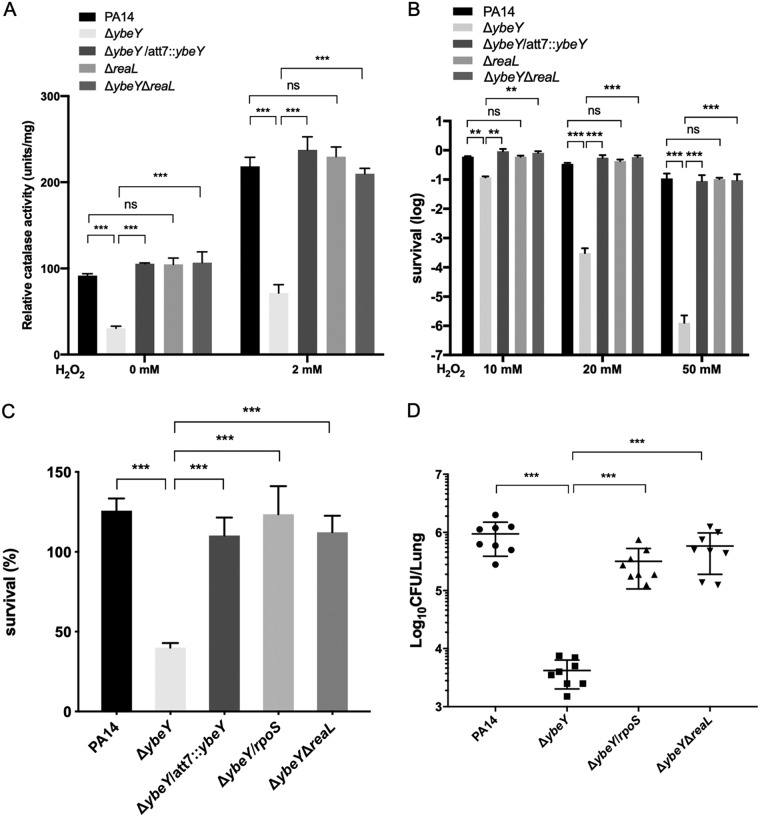
YbeY regulates the bacterial response to oxidative stresses and virulence through ReaL and RpoS. (A) The indicated strains were grown in LB to an OD_600_ of 1 and then incubated with or without 2 mM H_2_O_2_ for 30 min. The bacteria were collected by centrifugation, and the cells were broken by sonication. The total intracellular catalase activity was measured using a catalase assay kit. (B) The indicated strains were grown in LB to an OD_600_ of 1, and the cells were washed three times with PBS and then incubated with 10, 20, or 50 mM H_2_O_2_ for 30 min. The bacteria were collected by centrifugation and resuspended with fresh LB, and the survival rates were determined by plating. (C) The indicated strains were incubated with dHL60 cells at an MOI of 5 in HBSS for 3 h. The bacterial survival rates were determined by plating. (D) Mice were infected intranasally with the indicated strains. At 12 h postinfection, lungs from the infected mice were isolated. The bacterial loads were determined by serial dilution and plating. ns, not significant; **, *P* < 0.01; ***, *P* < 0.001 by Student’s *t* test.

Next, we examined whether the upregulation of ReaL contributes to the defective oxidative response and attenuated virulence of the Δ*ybeY* mutant. Deletion of *reaL* in the Δ*ybeY* mutant restored the total catalase activity (Fig. 10A) as well as the bacterial tolerance to H_2_O_2_ (Fig. 10B), whereas deletion of *reaL* in the wild-type PA14 did not affect these phenotypes. In addition, overexpression of *rpoS* or deletion of *reaL* in the Δ*ybeY* mutant restored the bacterial survival rate after incubation with dHL60 cells (Fig. 10C). We further examined the bacterial virulence in the acute pneumonia model. Overexpression of *rpoS* or deletion of *reaL* in the Δ*ybeY* mutant restored the bacterial loads in the lungs (Fig. 10D), whereas deletion of *reaL* in the wild-type PA14 did not affect the bacterial loads in the lungs (date not shown). Deletion of *reaL* in the Δ*ybeY* mutant did not affect the bacterial growth rate, while overexpression of RpoS slightly increased the growth rate (Fig. S5). In combination, these results indicate that the ReaL-mediated downregulation of RpoS and subsequent defective oxidative stress response contribute to the attenuated virulence of the Δ*ybeY* mutant.

### Mutation of *ybeZ* results in similar phenotypes as those of the Δ*ybeY* mutant.

**FIG 11:**
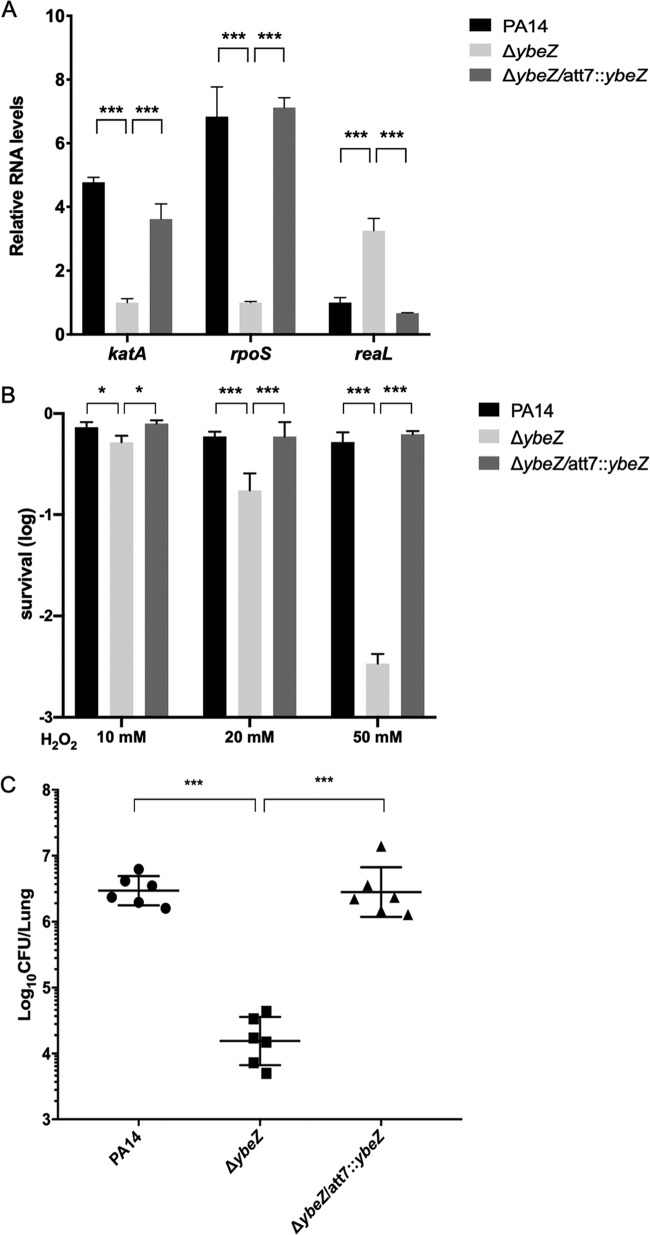
Roles of YbeZ in the bacterial response to oxidative stress and virulence. (A) Wild-type PA14, the Δ*ybeZ* mutant, and the complemented strain were grown in LB to an OD_600_ of 1. The relative RNA levels of *katA*, *rpoS*, and *reaL* were determined by real-time PCR. Results represent means ± SD. (B) The indicated strains were grown in LB to an OD_600_ of 1. Then the cells were washed three times with PBS and incubated with 10, 20, or 50 mM H_2_O_2_ for 30 min. The bacteria were collected by centrifugation and resuspended with fresh LB, and the survival rates were determined by plating. (C) Mice were infected intranasally with the indicated strains. At 12 h postinfection, lungs from the infected mice were isolated. The bacterial loads were determined by serial dilution and plating. *, *P* < 0.05; ***, *P* < 0.001 by Student’s *t* test.

As we found that YbeZ binds to YbeY and is involved in the maturation of the 16S rRNA, it is likely that YbeZ plays similar roles as YbeY. Indeed, mutation of *ybeZ* resulted in upregulation of *reaL* and downregulation of *rpoS* and *katA* (Fig. 11A). Consistently, the Δ*ybeZ* mutant was more susceptible to H_2_O_2_ and displayed attenuated virulence in the acute pneumonia model (Fig. 11B and C). These results suggest that YbeY and YbeZ are likely to function together in the regulation of *reaL*, *rpoS*, and oxidative stress response genes.

## DISCUSSION

YbeY is a highly conserved bacterial RNase that is involved in the 16S rRNA maturation, ribosome assembly, virulence, and stress responses (28–33). In previous studies, the *ybeY* mutant was not identified from P. aeruginosa transposon mutant libraries. Thus, *ybeY* was predicted to be an essential gene (29, 39). In this study, we were able to delete *ybeY* in the backgrounds of PA14, PAO1, and PAK. However, it took at least 2 days for the Δ*ybeY* mutant to form visible colonies on the LB plate. We suspect that the growth defect might explain why the *ybeY* mutant was not identified from the Tn mutant population. Mutation of *ybeY* resulted in defective processing of the 16S rRNA, defective response to oxidative stresses, and attenuated virulence in the murine acute pneumonia model. In addition, we found that the *ybeY* mutant was more susceptible to heat shock (45°C, data not shown).

Previous studies in E. coli identified YbeY as a metal-dependent hydrolase, and the three-dimensional crystal structure of the YbeY revealed a conserved metal ion binding pocket (40). Deletion of *ybeY* in E. coli reduces protein translation efficiency by affecting the 30S ribosome subunit. Davies et al. reported that YbeY is involved in the maturation of 16S rRNA (30). Jacob et al. demonstrated that YbeY is a single-stranded RNA (ssRNA) specific endoribonuclease and plays key roles in the ribosome quality control and 16S rRNA maturation together with RNase R in E. coli (28). A structural model of the E. coli YbeY revealed a positively charged cavity similar to the middle domain of Argonaute (AGO) proteins involved in RNA silencing in eukaryotes (51). Recent studies in E. coli, Sinorhizobium meliloti, and Vibrio cholerae demonstrated that the defect in YbeY results in aberrant expression of small RNAs (sRNAs) and the corresponding target mRNAs (29, 51, 52). The YbeY purified from S. meliloti displays a metal-dependent endoribonuclease activity that cleaves both ssRNA and double-stranded RNA (dsRNA) substrates (33).

A previous bacterial two-hybrid analysis revealed that YbeY interacts with the ribosomal protein S11, Era, Der, SpoT, and YbeZ in E. coli (37). The interaction between YbeY and S11 is required for the maturation of the 16S rRNA (37). Overexpression of the GTPase gene *era* in a Δ*ybeY* mutant improves the 16S rRNA maturation and 70S ribosome assembly (53). SpoT plays an important role in the bacterial stringent response by controlling the homeostasis of the alarmone molecule (p)ppGpp (54). Of note, (p)ppGpp controls the bacterial growth rate by suppressing the rRNA production (55). Further studies are warranted to explore whether YbeY affects the (p)ppGpp level and thus the bacterial stringent response. The GTPase Der (double Era-like GTPase) contains two GTP-binding domains. Studies in E. coli demonstrated its essentiality in the biogenesis of the 50S ribosomal subunit (56). In bacteria, the colocalization of *ybeZ* and *ybeY* in an operon is highly conserved (36–38). Here, we demonstrated the interaction between YbeY and YbeZ in P. aeruginosa and found that mutation of *ybeZ* resulted in similar phenotypes as the *ybeY* mutant. Further studies are needed to understand the exact function of YbeZ.

Here, we found that YbeY affects the expression of RpoS. A comparison of the transcription profiles between the *ybeY* mutant and an *rpoS* mutant revealed that 49 genes displayed similar expression patterns in the two mutants compared to the wild-type strain (see Table S2 in the supplemental material) (57). For example, previous studies revealed that the P. aeruginosa lectin PA-IL coding gene *lecA* and the two-component response regulator gene *pprB* are under the direct regulation of RpoS (58, 59). Mutation of *rpoS* resulted in downregulation of *lecA*, *pprB*, and genes regulated by the PprB, including the *tad* locus (PA4297-PA4305) and the type IVb pilin gene *flp* (59). Mutation of *ybeY* also reduced the expression of those genes (Table S2). However, 7 genes were oppositely expressed in the *ybeY* mutant and the *rpoS* mutant (Table S2), and many other genes displayed different expression patterns in the two mutants. These results indicate that YbeY controls global gene expression through multiple pathways.

In P. aeruginosa, RpoS controls the expression of the catalase genes *katA*/*B* (18); however, no RpoS binding sequence has been identified in the promoter regions of the two genes, indicating an indirect regulation (47). Overexpression of *rpoS* in the Δ*ybeY* mutant restored the expression levels of *katA* (Fig. 6) and *sodB* (data not shown). However, overexpression of *rpoS* only restores the expression level of *katB* in the Δ*ybeY* mutant in the absence of H_2_O_2_, but not in the presence of H_2_O_2_ (data not shown). The RpoS-mediated regulatory pathways on *katA*, *katB*, and *sodB* warrant further studies.

Upon invading host, the bacteria encounter phagocytes that kill the bacteria mainly through ROS, phagocytosis, antimicrobial peptides, and hydrolases (12, 60, 61). Bacteria survive the phagocyte-generated ROS by producing superoxide dismutase and catalases (13, 62). Our *in vitro* infection assay revealed that the *ybeY* mutant was more susceptible to neutrophils. Neutralization of ROS by NAC restored the bacterial survival rate, indicating that the hypersusceptibility to neutrophil is mainly due to defective detoxification of the ROS. Therefore, the downregulation of *katA* might partially contribute to the attenuated virulence of the *ybeY* mutant. Meanwhile, RpoS controls multiple stress response genes and affects the quorum sensing systems (57). The defective processing of rRNA might also affect the bacterial adaptation to the host environment and expression of virulence factors. Comparing the gene expression profile between the *ybeY* mutant and the wild-type strain during infection might shed light on the roles of YbeY in the bacterial virulence.

Overall, our study reveals an important physiological role of YbeY in the RNA processing in P. aeruginosa. Further studies are required to understand how the expression and activity of YbeY are regulated, particularly under environmental stresses.

## MATERIALS AND METHODS

### Bacterial strains and plasmids.

The bacterial strains, plasmids, and primers used in this study are listed in Table S1 in the supplemental material. Bacteria were cultured in the L-broth (63) medium at 37°C with agitation at 200 rpm. Antibiotics were used at the following concentrations: for E. coli, gentamicin 10 μg ml^−1^, tetracycline 10 μg ml^−1^, kanamycin 50 μg ml^−1^, ampicillin 100 μg ml^−1^; for P. aeruginosa, tetracycline 50 μg ml^−1^, gentamicin 50 μg ml^−1^, rifampin 100 μg ml^−1^, carbenicillin 150 μg ml^−1^. Chromosomal gene mutations were generated as described previously (64). To construct a *ybeY* deletion mutant in P. aeruginosa, a 945-bp fragment and a 1,306-bp fragment that are upstream and downstream of the *ybeY* coding region, respectively, were amplified by PCR using PA14 chromosome as the template and primers listed in Table S1. The fragments were cloned into the plasmid pEX18TC. The resulting plasmid was transferred into E. coli S17 and then transferred into P. aeruginosa by conjunction. Single crossover mutants were selected on 50 μg ml^−1^ tetracycline and 50 μg ml^−1^ kanamycin (to kill the E. coli donor strain), and double crossover mutants were selected by growth on LB plates containing 7.5% sucrose. The *ybeY* deletion mutant was screened by PCR with the primers YbeY-L and YbeY-R (Table S1). In the *ybeY* deletion mutant, a 421-bp fragment was deleted from the 483-bp coding region of the *ybeY* gene.

10.1128/mBio.00659-20.8 TABLE S1 Bacterial strains, plasmids, and primers used in this study. Download Table S1, DOCX file, 0.04 MB. Copyright © 2020 Xia et al. 2020 Xia et al. This content is distributed under the terms of the Creative Commons Attribution 4.0 International license.

10.1128/mBio.00659-20.9 TABLE S2 RNA-seq results of Δ*ybeY* and PA14. Genes that displayed similar expression patterns in the *ybeY* and *rpoS* mutants in comparison to the wild-type strain are shown in red, and those with opposite expression patterns are shown in blue. Download Table S2, DOCX file, 0.03 MB. Copyright © 2020 Xia et al. 2020 Xia et al. This content is distributed under the terms of the Creative Commons Attribution 4.0 International license.

### Transcriptome sequencing and analysis.

Bacteria were cultured in LB at 37°C to the stationary phase (OD_600_ = 3). Total RNA was extracted by the RNA prep Pure Cell/Bacteria kit (Tiangen Biotec, Beijing, China). Sequencing and analysis services were performed by the Suzhou Genewiz as previously described (65).

### Real-time PCR.

Bacterial cells were cultured under indicated conditions. Total bacterial RNA was extracted by an RNA prep Pure Cell/Bacteria kit (Tiangen Biotec, Beijing, China). cDNAs were synthesized using random primers and reverse transcriptase (TaKaRa, Dalian, China). Real-time PCR was performed with the SYBR II Green supermix (TaKaRa, Dalian, China). The ribosomal gene *rpsL* or PA1805 was used as the internal control (66).

### rRNA analysis.

Bacteria were cultured in LB at 37°C until late logarithmic phase (OD_600_ = 1). Total RNA was isolated with the RNA prep Pure Cell/Bacteria kit (Tiangen Biotec, Beijing, China). One microgram of the total RNA was mixed with an RNA loading buffer (TaKaRa, Dalian, China) and incubated at 65°C for 10 min, followed by incubation on ice for 10 min. Then the 16S and 23S rRNAs were separated by electrophoresis on a gel made of 0.9% Synergel (BioWorld, USA) and 0.7% agarose in TAE as described previously (67).

### Ribosome separation by sucrose gradients.

Separation of P. aeruginosa ribosomal particles was performed as previously described (41) with minor modifications. Bacteria were grown in LB at 37°C to an OD_600_ of 1. One liter of the bacterial cells was collected and resuspended in 40 ml precooled Buffer A (10 mM Tris–HCl, 10 mM MgCl_2_, 100 mM NH_4_Cl, 6 mM β-mercaptoethanol, pH 7.5) with 10 μg/ml DNase I. The bacterial cells were lysed by a French press (600 MPa), and the cell debris was removed by centrifugation at 12,000 × *g* for 30 min at 4°C. One milliliter of the supernatant was loaded onto a 10% to 40% sucrose gradient in buffer A, followed by centrifugation at 36,000 rpm for 3 h at 4°C in an SW41 rotor (Beckman). The stratified sucrose gradient solutions were collected, and the RNA contents were quantified by UV absorbance at 260 nm.

### Pulldown assay.

The *ybeY* and *ybeZ* coding regions were amplified by PCR using PA14 chromosome DNA as the template, and the primers are listed in Table S1. A 6×His tag coding sequence was included in the downstream primer of the *ybeY* gene (Table S1), thus resulting in a C-terminal 6×His-tagged *ybeY* (*ybeY*-His). The amplified *ybeY* gene was cloned into the plasmid pMMB67EH. The *ybeZ* gene was cloned into the plasmid pET41a, resulting in a translational fusion with the *gst* gene at the C terminus. E. coli strain BL21 carrying the *gst* gene or *ybeZ*-*gst* fusion gene was cultured at 37°C to an OD_600_ of 0.4 to 0.6. Expression of the YbeZ-GST was induced by 1 mM IPTG for 4 h, and then the bacteria were collected and resuspended in a lysis buffer (1 M Na_2_HPO_4_, 1 M NaH_2_PO_4_, 0.3 M NaCl, pH 8.0), followed by sonication. The cell debris was removed by centrifugation at 12,000 × *g* for 20 min at 4°C. The cell lysate containing GST or YbeZ-GST protein was incubated with the GST tag purification resin (Beyotime Biotechnology, Shanghai, China) for 2 h at 4°C and then washed three times with the lysis buffer. PA14 carrying the *ybeY*-His fusion gene was grown in LB to an OD_600_ of 0.6, followed by induction by 1 mM IPTG for 4 h. The bacteria were resuspended in the lysis buffer and lysed by sonication. The cell lysate was then incubated with the GST or YbeZ-GST bound resin at 4°C for 2 h. The resin was washed three times with the lysis buffer. The bound proteins were then eluted with 60 mM reduced glutathione (GSH) in an elution buffer provided by the purification kit (Beyotime Biotechnology, Shanghai, China) and subjected to Western blot analysis.

### β-Galactosidase assay.

Bacterial cells were grown in LB at 37°C until the OD_600_ reached 1. An 0.5-ml portion of the bacterial culture was collected and resuspended in 1.5 ml Z buffer (50 mM β-mercaptoethanol, 60 mM Na_2_HPO_4_, 60 mM NaH_2_PO_4_, 10 mM KCl, 1 mM MgSO_4_, pH 7.0). The β-galactosidase activity was determined as previously described (68).

### H_2_O_2_ susceptibility assay.

Bacteria were cultured to an OD_600_ of 1 and then washed three times with PBS and resuspended in PBS. The bacteria were treated with 10, 20, or 50 mM H_2_O_2_ at 37°C for 30 min. The live bacterial numbers were determined by serial dilution and plating. The survival rate was calculated by comparing the live bacterial number after the H_2_O_2_ treatment with that before the treatment.

### Catalase activity assay.

Bacteria were cultured to an OD_600_ of 1 and then incubated with or without 2 mM H_2_O_2_ for 30 min. The bacteria were collected by centrifugation, and the cells were broken by sonication. The total intracellular catalase activity was measured using a catalase assay kit (Beyotime, Shanghai, China). The total bacterial protein concentrations were quantified by a BCA analysis (Beyotime, Shanghai, China) for calibration.

### HL60 differentiation and killing assay.

HL60 cells were cultured in RPMI 1640 medium (HyClone, USA), with streptomycin (100 mg/ml), penicillin G (100 U/ml), and 10% (vol/vol) thermally inactivated fetal bovine serum (Gibco, Australia) at 37°C with 5% CO_2_. The HL60 cells were diluted to 4.5 × 10^5^ cells/ml and cultured in the differentiation medium (RPMI 1640 medium, 20% [vol/vol] heat-inactivated fetal bovine serum, 1.3% dimethyl sulfoxide [Sigma, USA]) for 6 to 7 days (20). The differentiated HL60 cells (designated dHL60) were diluted to 1 × 10^7^ cells/ml in warm HBSS, and 100 μl cell suspension was added to each well of a 96-well plate. Then the cells were infected with bacteria at an MOI of 10 and incubated at 37°C for 3 h. The live bacterial numbers were determined by serial dilution and plating. The survival rate was calculated by comparing the numbers of live bacterial cells after incubation with or without the dHL60 cells.

### RNA stability analysis.

P. aeruginosa strains were cultured in LB at 37°C to an OD_600_ of 1. The bacterial cultures were then treated with 100 μg/ml rifampin to stop the transcription. At the indicated time points, the bacterial cells were collected and mixed with the same number of E. coli cells expressing a *gfp* gene. Total RNA was purified, and the levels of *reaL* and *rpsL* were determined by real-time PCR. The *gfp* mRNA levels were used as the internal control for normalization.

### *In vitro* transcription and RNA gel mobility shift assay.

The *in vitro* transcription of sRNA was performed as previously described (22). The sRNA ReaL was synthesized using the Riboprobe System-T7 (Promega) from PCR product amplified from PA14 chromosomal DNA with the primers listed in Table S1 according to the manufacturer’s instructions. The RNA was purified by isopropanol precipitation and refolded by heating at 90°C for 10 min and then cooling down naturally at room temperature for 30 min. One hundred nanograms of the purified RNA was mixed with indicated amounts of purified YbeY or GFP in the binding buffer (10 mM Tris-HCl [pH 7.5], 5 mM MgCl_2_, 50 mM KCl, 10% glycerol, 1 U recombinant RNase inhibitor [TaKaRa]) and incubated on ice for 30 min. Fifteen microliters of each sample was loaded onto a nondenaturing 8% polyacrylamide gel. The electrophoresis was performed at 100 V for 150 min in 0.5× TBE buffer on ice. The RNA bands were visualized after staining with Gel-red (Biotium) for 10 min.

### *In vitro* RNA degradation assay.

The sRNA ReaL was synthesized using a Riboprobe System-T7 (Promega) from the PCR product amplified from PA14 chromosomal DNA with the primers listed in Table S1. The RNA was purified by isopropanol precipitation and refolded by heating at 90°C for 10 min and then cooling down naturally at room temperature for 30 min. One hundred nanograms of the purified RNA was mixed with indicated amounts of the purified YbeY or GFP protein in the activity buffer (10 mM Tris-HCl [pH 7.5], 50 mM KCl, 10% glycerol, and the indicated concentrations of MnCl_2_) and incubated at 37°C for 30 min. Fifteen microliters of each sample was loaded onto a nondenaturing 8% polyacrylamide gel. Electrophoresis was performed at 100 V for 150 min in 0.5× TBE buffer on ice. The RNA was visualized after staining with Gel-red (Biotium) for 10 min.

### Murine acute pneumonia model.

The animal infection experiments described in this study were performed following the National and Nankai University guidelines on the use of animals in research. The protocol with the permit number NK-04-2012 was approved by the animal care and use committee of the College of Life Sciences, Nankai University. The infection was performed as previously described (69). Briefly, bacteria were cultured overnight and then diluted at 1:100 in fresh LB and grown at 37°C with agitation until OD_600_ reached 1. The bacterial cells were harvested by centrifugation, washed once with PBS, and resuspended in PBS at the concentration of 2 × 10^8^ CFU/ml. Each 6- to 8-week-old female BALB/c mouse (Vital River, Beijing, China) was anesthetized by an intraperitoneal injection of 100 μl 7.5% chloral hydrate, followed by an intranasal inoculation of 20 μl of the bacterial suspension, resulting in 4 × 10^6^ CFU per mouse. The mice were sacrificed 12 h postinfection, and the lungs were dissected and subjected to homogenization. The number of bacteria in each lung was determined by plating.

### Data availability.

The transcriptome (RNA sequencing) data that support the findings of this study have been deposited in the NCBI Sequence Read Archive (SRA) with the accession code PRJNA574019.
