# Supplementary material for: Endoribonuclease YbeY Is Essential for RNA Processing and Virulence in Pseudomonas aeruginosa
Source: mBio. 2020 Jun 30;11(3):e00659-20. doi: 10.1128/mBio.00659-20 (PMC7327168; doi:10.1128/mBio.00659-20)
Supplement: FIG S1 [file mBio.00659-20-sf001.pdf]

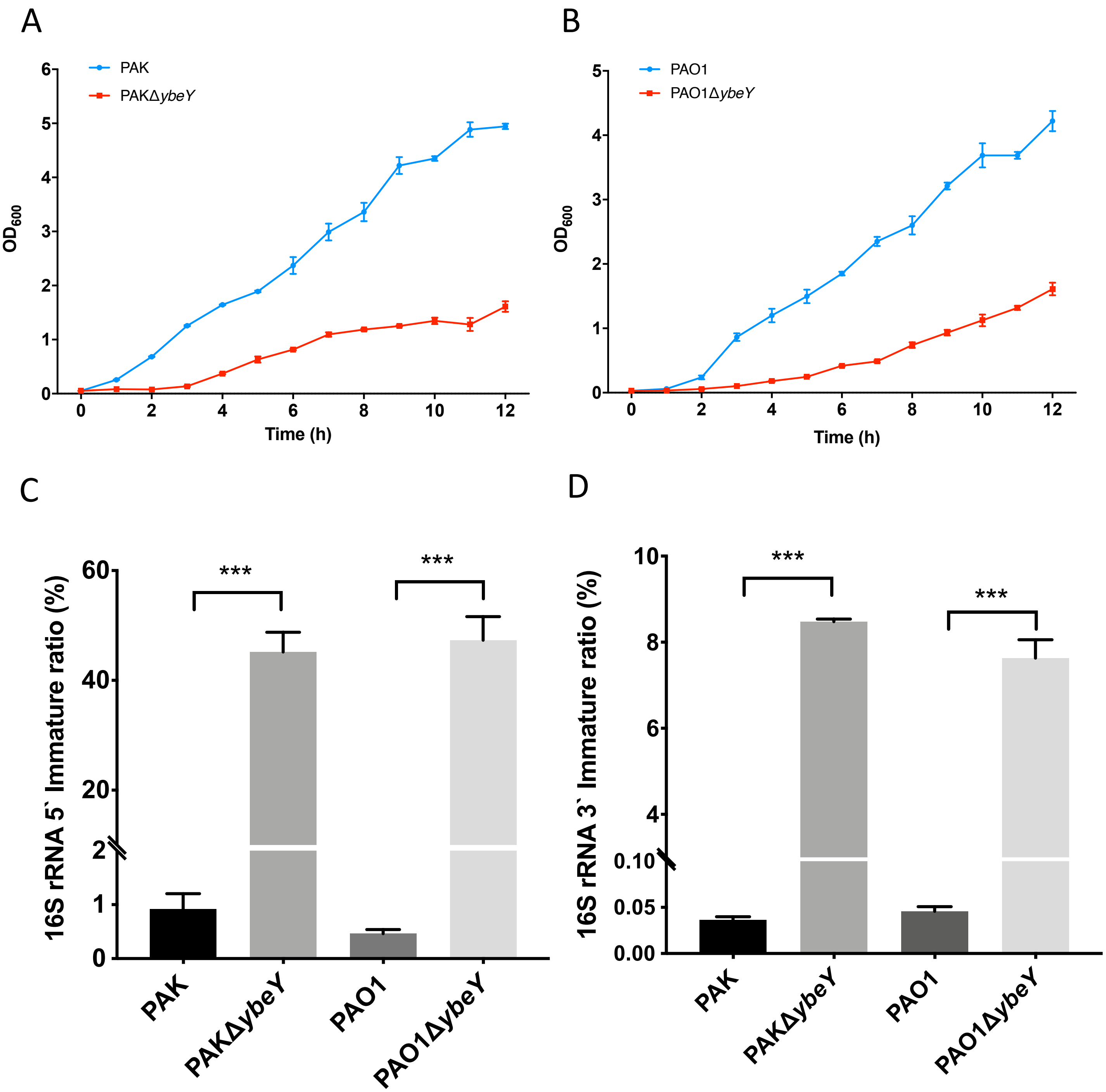

Fig. S1 YbeY influences the growth rate and 16S rRNA maturation in PAO1 and PAK (A, B) Bacterial growth rates. Same number of cells of the indicated strains were inoculated in LB. The OD_{600} was monitored every hour for 12 hours. (C, D) The bacterial total RNA was isolated and the 5'-and 3'-immature ratios of the 16S rRNA was determined by real-time PCR. ***, $P < 0.001$ by Student's t-test.
