# Supplementary material for: Endoribonuclease YbeY Is Essential for RNA Processing and Virulence in Pseudomonas aeruginosa
Source: mBio. 2020 Jun 30;11(3):e00659-20. doi: 10.1128/mBio.00659-20 (PMC7327168; doi:10.1128/mBio.00659-20)
Supplement: FIG S2 [file mBio.00659-20-sf002.pdf]

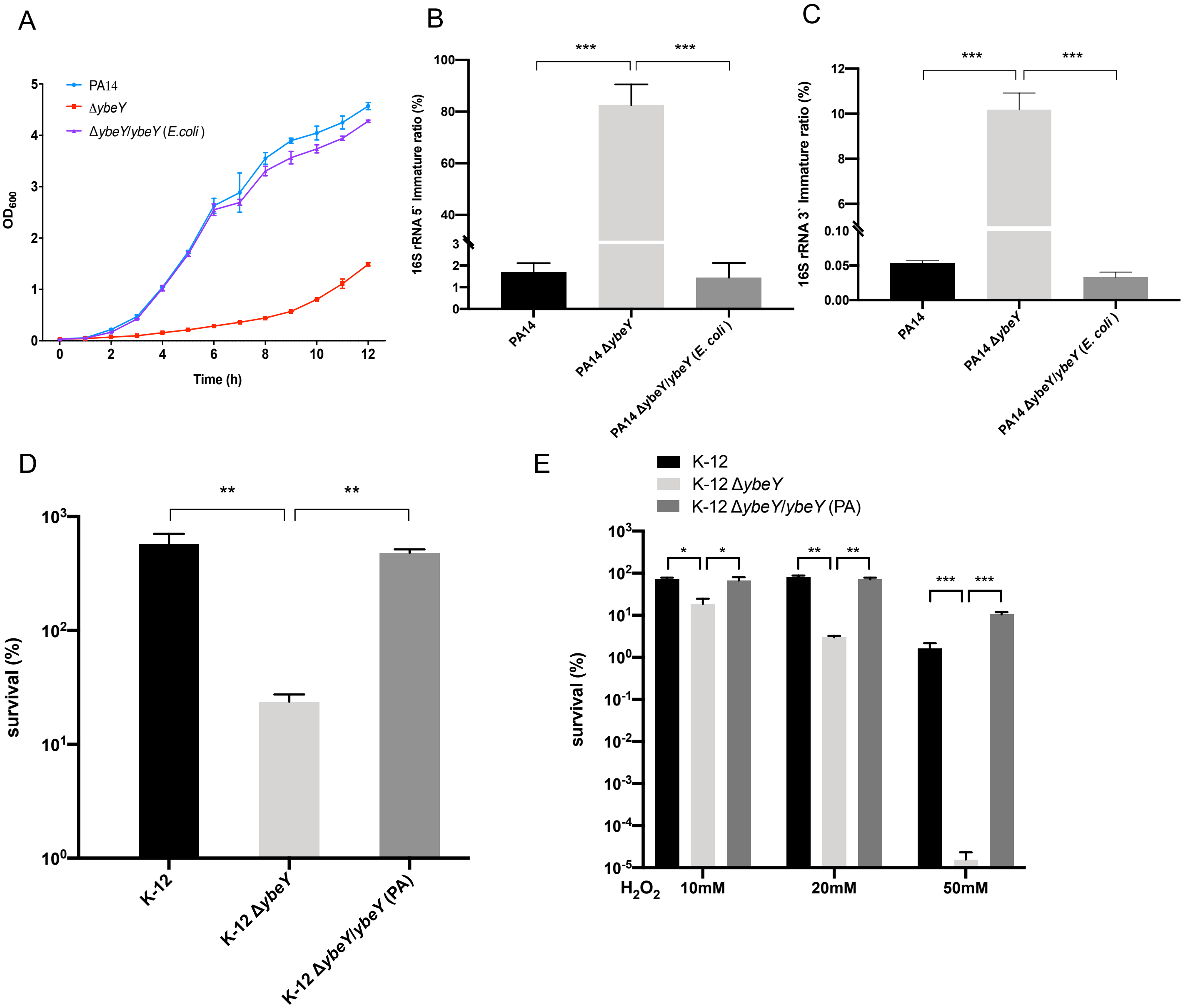

Fig. S2 Functional similarities between the *P. aeruginosa* and *E.coli* YbeY. (A) Same number of cells of the indicated strains were inoculated in LB. The OD_{600} was monitored every hour for 12 hours. (B, C) The bacteria were grown to an OD_{600} of 1.0, followed by total RNA isolation. The 5'-and 3'-immature ratios of the 16S rRNA was determined by real-time PCR. (D) The bacteria were grown to an OD_{600} of 1.0 at 37 ºC and then incubated at 45 ºC for two hours. The live bacterial cell numbers were determined by serial dilution and plating. (E) The bacteria were cultured to an OD_{600} of 1, and then washed three times with PBS and resuspended in PBS. The bacteria were treated with 10, 20 or 50 mM H_2O_2 at 37 ºC for 30 min. The live bacterial cell numbers were determined by serial dilution and plating. The survival rate was calculated by comparing the live bacterial number after the treatment with that before the treatment. * $P < 0.05$, ** $P < 0.01$, *** $P < 0.001$ by Student's t-test.
