# Supplementary material for: Endoribonuclease YbeY Is Essential for RNA Processing and Virulence in Pseudomonas aeruginosa
Source: mBio. 2020 Jun 30;11(3):e00659-20. doi: 10.1128/mBio.00659-20 (PMC7327168; doi:10.1128/mBio.00659-20)
Supplement: FIG S3 [file mBio.00659-20-sf003.pdf]

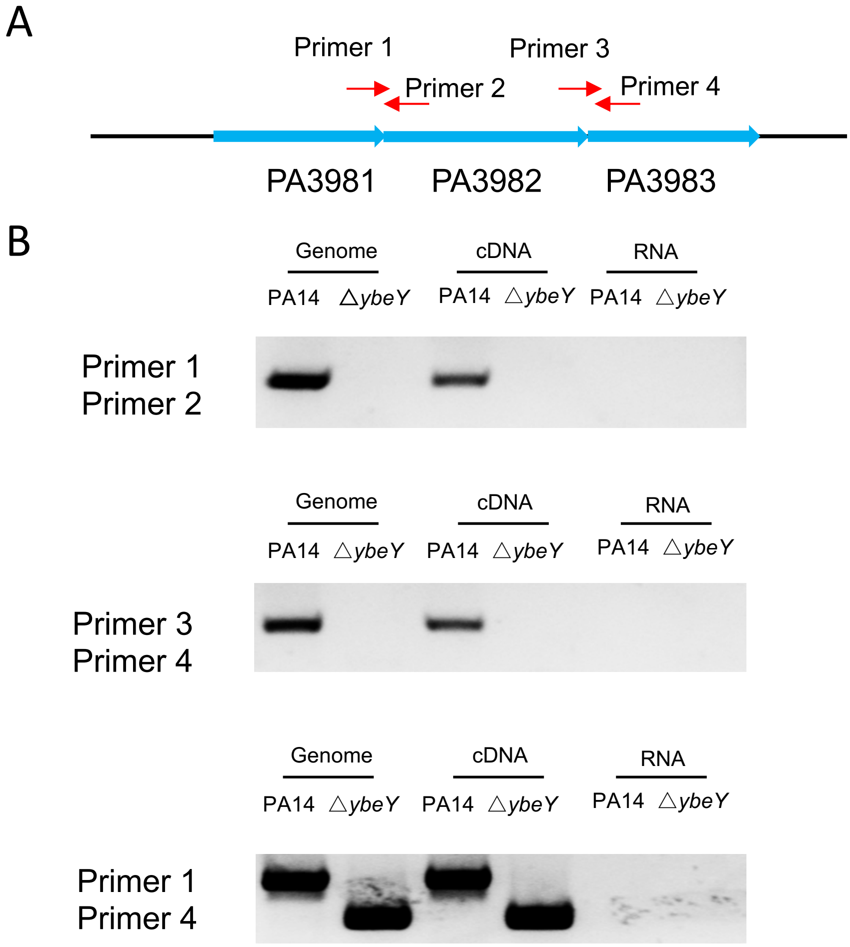

Fig. S3 PA3981, PA3982 and PA3983 are in one operon. (A) Locations of the primers. (B) PCR products from indicated primers and templates.
