# Supplementary material for: Endoribonuclease YbeY Is Essential for RNA Processing and Virulence in Pseudomonas aeruginosa
Source: mBio. 2020 Jun 30;11(3):e00659-20. doi: 10.1128/mBio.00659-20 (PMC7327168; doi:10.1128/mBio.00659-20)
Supplement: FIG S4 [file mBio.00659-20-sf004.pdf]

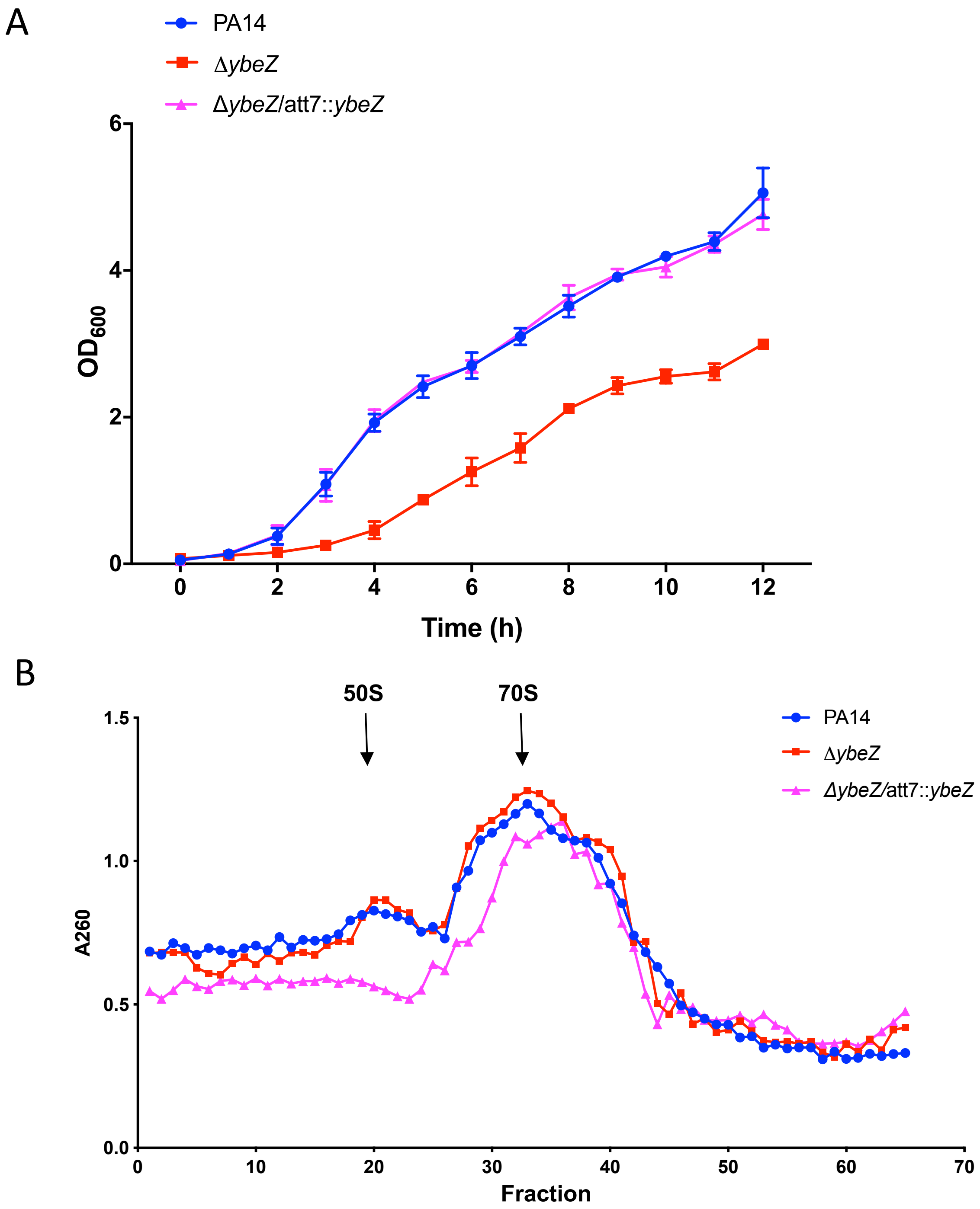

Fig. S4. Roles of YbeZ in the bacterial growth and ribosome assemble. (A) Growth rate of the indicated strains in LB medium. Overnight cultures of indicated strains were 1:100 diluted into fresh LB. The bacterial growth was monitored by measuring OD_{600} every hour for 12 hours. (B) Ribosome profiles from PA14, the Δ*ybeZ* and Δ*ybeZ*/att7::*ybeZ* strains. The bacteria were grown to an OD_{600} of 1. The ribosome particles were subjected to sucrose gradient separation and quantified by UV absorbance at 260 nm.
