# Supplementary material for: Endoribonuclease YbeY Is Essential for RNA Processing and Virulence in Pseudomonas aeruginosa
Source: mBio. 2020 Jun 30;11(3):e00659-20. doi: 10.1128/mBio.00659-20 (PMC7327168; doi:10.1128/mBio.00659-20)
Supplement: FIG S5 [file mBio.00659-20-sf005.pdf]

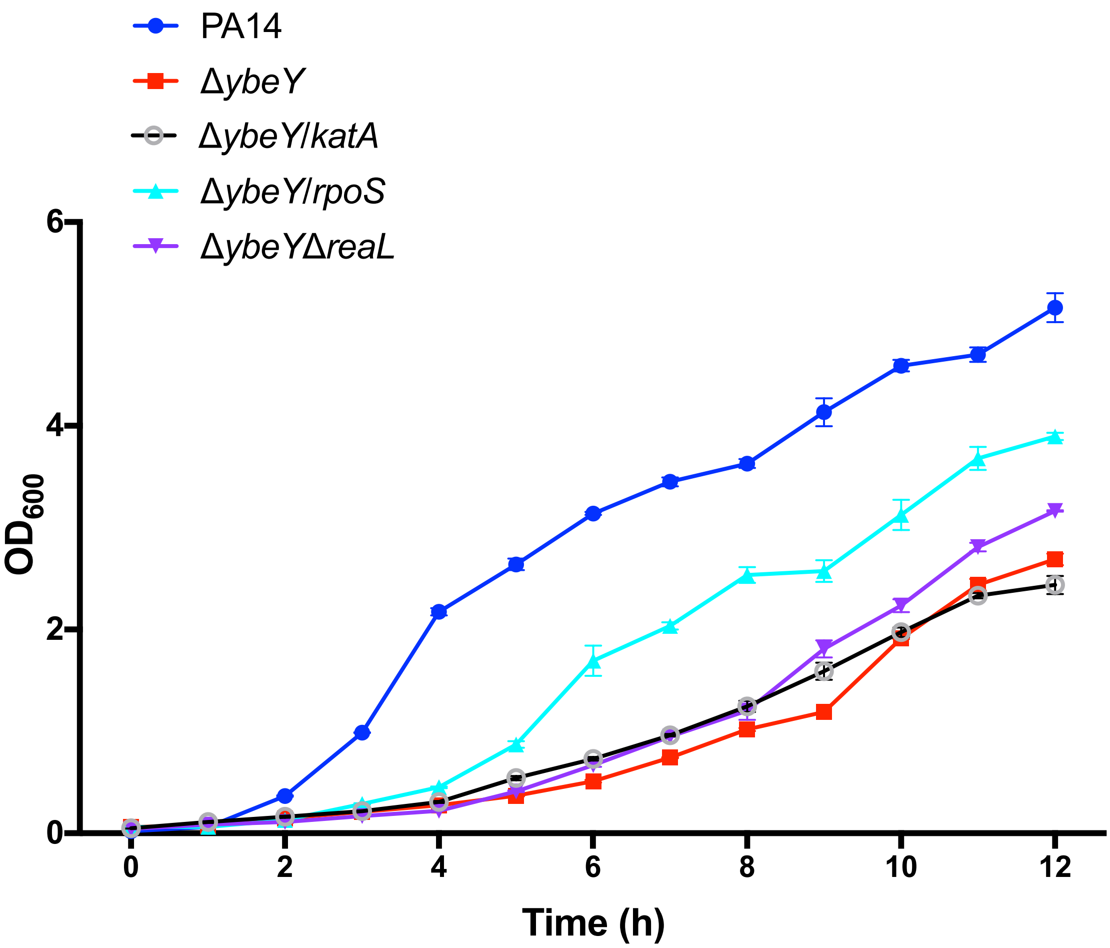

Fig. S5. Growth rate of indicated strains in LB medium. Overnight cultures of the indicated strains were 1:100 diluted into fresh LB. The bacterial growth was monitored by measuring OD_{600} every hour for 12 hours.
