# Supplementary material for: Endoribonuclease YbeY Is Essential for RNA Processing and Virulence in Pseudomonas aeruginosa
Source: mBio. 2020 Jun 30;11(3):e00659-20. doi: 10.1128/mBio.00659-20 (PMC7327168; doi:10.1128/mBio.00659-20)
Supplement: FIG S6 [file mBio.00659-20-sf006.pdf]

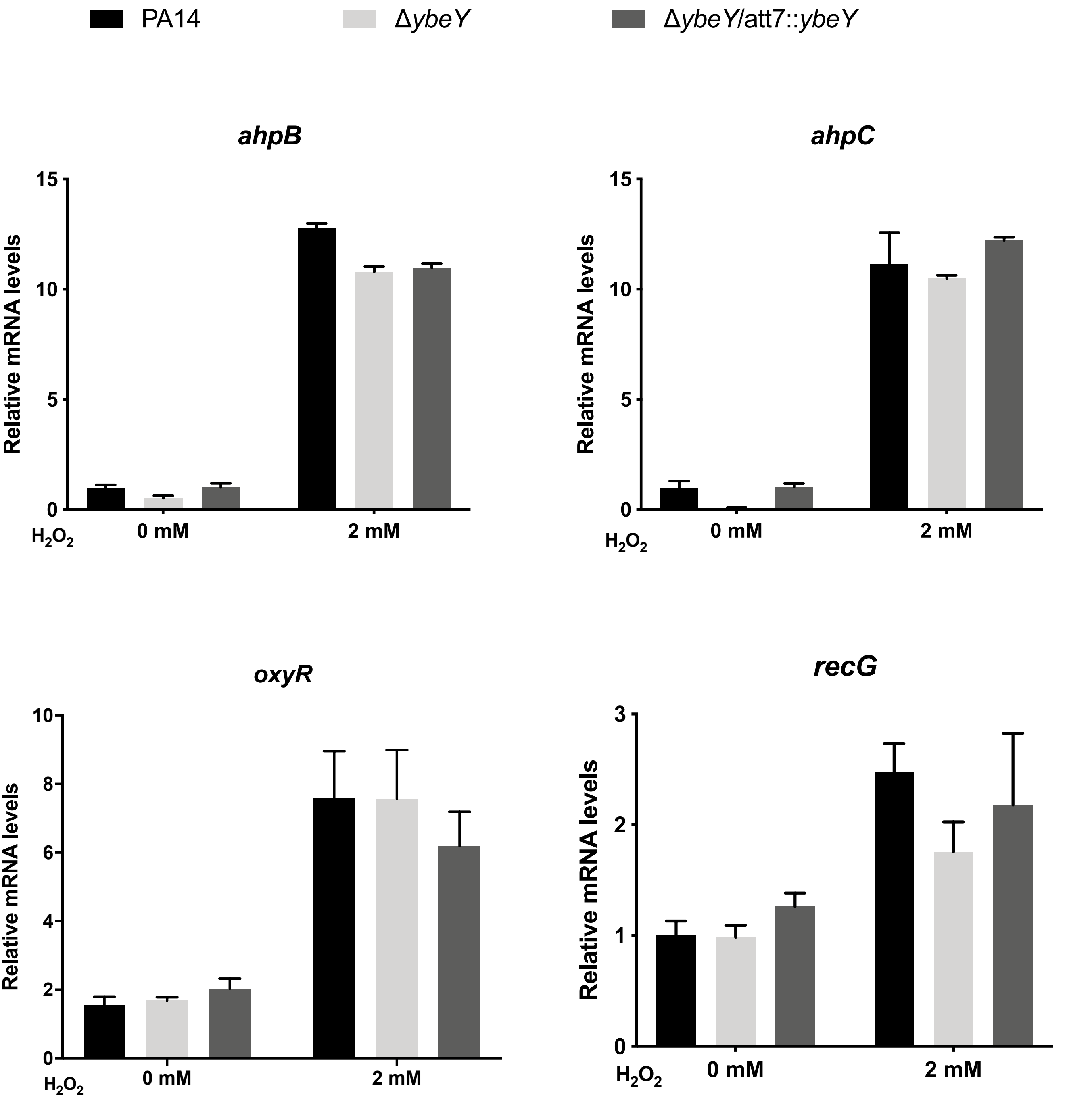

Fig. S6. The expression of oxidative stresses response related genes. Wild type PA14, the Δ*ybeY* mutant and the complemented strain were grown in LB to an OD_{600} of 1, then incubated with or without 2 mM H_2O_2 for 30 min. The relative mRNA levels were determined by real time PCR. Results represent means ± SD.
