# Supplementary material for: Endoribonuclease YbeY Is Essential for RNA Processing and Virulence in Pseudomonas aeruginosa
Source: mBio. 2020 Jun 30;11(3):e00659-20. doi: 10.1128/mBio.00659-20 (PMC7327168; doi:10.1128/mBio.00659-20)
Supplement: FIG S7 [file mBio.00659-20-sf007.pdf]

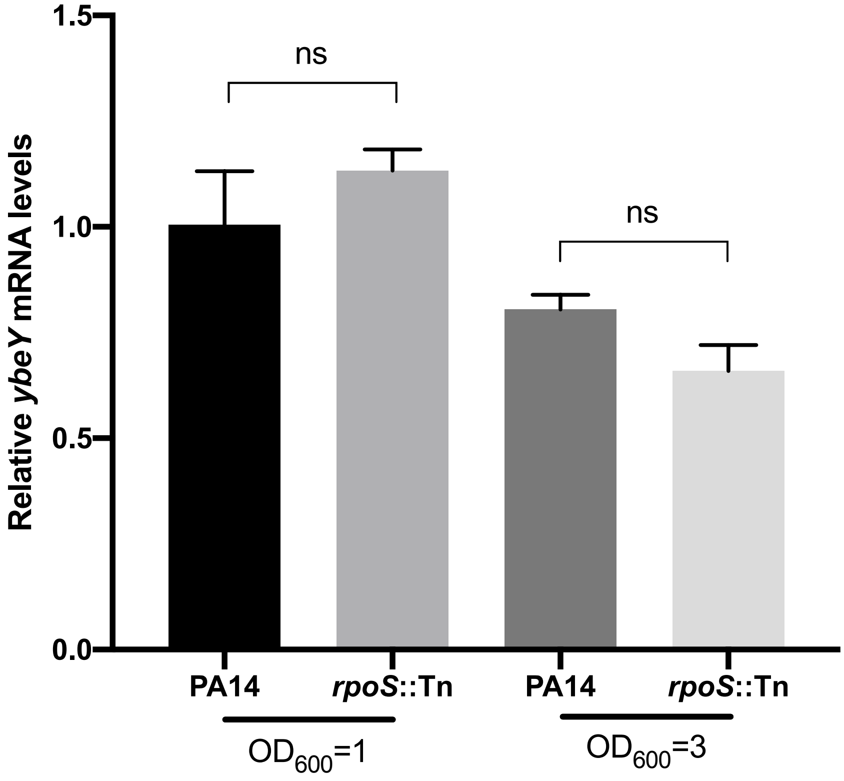

Fig. S7. The expression of *ybeY*. Wild type PA14 and the *rpoS*::Tn mutant were grown in LB to an OD_{600} of 1 or 3. The relative mRNA levels of *ybeY* were determined by real time PCR. Results represent means ± SD. ns, not significant by Student's t-test.
