# Supplementary material for: Endoribonuclease YbeY Is Essential for RNA Processing and Virulence in Pseudomonas aeruginosa
Source: mBio. 2020 Jun 30;11(3):e00659-20. doi: 10.1128/mBio.00659-20 (PMC7327168; doi:10.1128/mBio.00659-20)
Supplement: TABLE S1 [file mBio.00659-20-st001.docx]

**Supplemental Material**

**Table S1. Bacterial strains, plasmids and primers used in this study.**

| **Strain/ Plasmid /Primer** | **Description** | **Source (Reference)** |
| --- | --- | --- |
| ***P. aeruginosa*** | | |
| PA14 | Wild type strain of *Pseudomonas aeruginosa* | ([1](#_ENREF_1)) |
| △*ybeY* | PA14 deleted of *ybeY* | This study |
| △*ybeY*/att7::*ybeY* | △*ybeY* with *ybeY* inserted on chromosome with mini-Tn7T insertion; GEN^r^ | This study |
| △*ybeY*/att7::*ybeY*/R56A | △*ybeY* with *ybeY*/R56A inserted on chromosome with mini-Tn7T insertion; GEN^r^ | This study |
| △*ybeY*/att7::*ybeY*/H112A | △*ybeY* with *ybeY*/H112A inserted on chromosome with mini-Tn7T insertion; GEN^r^ | This study |
| △*ybeY*/att7::*ybeY*/R56A/H112A | △*ybeY* with *ybeY*/R56A/H112A inserted on chromosome with mini-Tn7T insertion; GEN^r^ | This study |
| △*ybeZ* | PA14 deleted of *ybeZ* | This study |
| △*ybeZ*/att7::*ybeZ* | △*ybeZ* with *ybeZ* inserted on chromosome with mini-Tn7T insertion; GEN^r^ | This study |
| △*ybeY*/pUCP20-*rpoS* | △*ybeY* with plasmid pUCP20-*ropS*; CAR^r^ | This study |
| △*ybeY*/pUCP20-*katA* | △*ybeY* with plasmid pUCP20-*katA*; CAR^r^ | This study |
| △*reaL* | PA14 deleted of *reaL* | This study |
| △*ybeY*△*reaL* | PA14 deleted of *ybeY* and *reaL* | This study |
| *rpoS*::Tn | PA14 with MAR2 × T7 transposon inserted at *rpoS*; GEN^r^ | ([1](#_ENREF_1)) |
| *rpoS*::Tn/pUCP20-*rpoS* | *rpoS*::Tn with plasmid pUCP20-*ropS*; GEN^r^ CAR^r^ | This study |
| **Plasmid** |  |  |
| pUCP20 | *Escherichia–Pseudomonas* shuttle vector without lac promoter; AMP^r^ | ([2](#_ENREF_2)) |
| pEX18Tc | Gene replacement vector; TET^r^, *oriT*^+^, *sacB*^+^ | ([2](#_ENREF_2)) |
| pMMB67EH | Expression vector with *tac* promoter; AMP^r^ | ([3](#_ENREF_3)) |
| pUC18T-mini-Tn7T-Gm    pRKaraRed | mini-Tn7 base vector from insertion into chromosome attTn7 site; GEN^r^  Expression vector with P*_BAD_* promoter; TET^r^ | ([2](#_ENREF_2))  ([4](#_ENREF_4)) |
| pDN19lacΩ | Promoterless lacZ fusion vector; SPT^r,^ STR^r^, TET^r^ | ([5](#_ENREF_5)) |
| pEX18Tc-△*ybeY*  pEX18Tc-△*ybeZ*  pUC18T-mini-Tn7T-Gm-*ybeY* | *ybeY* gene of PA14 deletion on pEX18Tc; TET^r^  *ybeZ* gene of PA14 deletion on pEX18Tc; TET^r^  pUC18T-mini-Tn7T-Gm with *ybeY*; GEN^r^ | This study  This study  This study |
| pUC18T-mini-Tn7T-Gm-*ybeZ*  pMMB67EH-*ybeY*-His | pUC18T-mini-Tn7T-Gm with *ybeZ*; GEN^r^  *ybeY* sequence of PA14 fused to His on pMMB67EH; AMP^r^ | This study  This study |
| pET41a-*ybeZ*-GST  pRKaraRed-*rpoS*(F3)-Flag | *ybeZ* sequence of PA14 fused to GST on pET41a; KAN^r^  *rpoS*(F3) fused to Flag on pRKaraRed; TET^r^ | This study  This study |
| pRKaraRed-*rpoS*(F2)-Flag  pRKaraRed-*rpoS*(F1)-Flag | *rpoS*(F2) fused to Flag on pRKaraRed; TET^r^  *rpoS*(F1) fused to Flag on pRKaraRed; TET^r^ | This study  This study |
| pRKaraRed-*rpoS*(RBS)-Flag | *rpoS*(RBS) fused to Flag on pRKaraRed; TET^r^ | This study |
| pUCP20-*katA*  pUCP20-*rpoS* | Overpression of *katA* on pUCP20; CAR^r^  Overpression of *rpoS* on pUCP20; CAR^r^ | This study  This study |
| **Primer** | **Sequence (5’→3’)** | **Function** |
| YbeY-L-F | TACTCAGAATTCCTCCACCAGATCGAGAAACG | *ybeY* deletion |
| YbeY-L-R | TACTCAGGATCCTGCAGGTCGAGCTCAAGC | *ybeY* deletion |
| YbeY-R-F  YbeY-R-R | TACTCAGGATCCTGCAGGTCGAGCTCAAGC  TACTCAAAGCTTGGCGCCGTAGTCATGGAT | *ybeY* deletion  *ybeY* deletion |
| YbeY-L | TACTCACCCGGGCCTTCATCTACAGCGCGC | *ybeY* cloning |
| YbeY-R | TACTCAAAGCTTTCATTTTTCCTTGCTCGGT | *ybeY* cloning |
| YbeZ-L-F | GCTCTAGATGGAAGGCTGCAGCAAGTAC | *ybeZ* deletion |
| YbeZ-L-R  YbeZ-R-F | CCCAAGCTTATCTCGATGTCGAGGCGTTT  CCCAAGCTTCTACGAGCGCCATGAGAACC | *ybeZ* deletion  *ybeZ* deletion |
| YbeZ-R-R | CGCGGATCCTGTTGTTGTGCCAATACATAAGC | *ybeZ* deletion |
| YbeZ-L | CCCAAGCTTCGGAAGAAGTGAAGAAACAGCG | *ybeZ* cloning |
| YbeZ-R | TCCCCCGGGGGAACTGCGCTTCGGAGG | *ybeZ* cloning |
| ReaL-L-F | CCCGAGCTCATTGTCCGGGGAAATCTACC | *reaL* deletion |
| ReaL-L-R | TGCTCTAGACATTTCGCATGAATCCTTTAGTT | *reaL* deletion |
| ReaL-R-F  ReaL-R-R | TGCTCTAGATCACCTTTTGCTCCTCTCGC  CCCAAGCTTCAGGACCCGGCTCTTCAGA | *reaL* deletion  *reaL* deletion |
| PMMB-YbeY-His-F | CCGGAATTCGTCGAGGGAACGGGCAAG | YbeY*-*His cloning |
| PMMB-YbeY-His-R | CCCAAGCTTTCAGTGGTGGTGGTGGTGGTGTTTTTCCTTGCTCGGTGGC | YbeY*-*His cloning |
| pET41a-YbeZ-F | GGACTAGTAACGCCCCCCAGGAACTT | YbeZ -GST cloning |
| pET41a-YbeZ-R  RpoS-Pro-F | CCCAAGCTTCGGAACTGTGCTTCGGAGG  TACTCAGAATTCCCTGCGAGCGGTAGTCTGAT | YbeZ -GST cloning  P*rpoS-*LacZ cloning |
| RpoS-Pro-R | TACTCAGGATCCAGTGGTGGCTTTTGGAGTTGC | P*rpoS*-LacZ cloning |
| ReaL-Pro-F | CCGGAATTCCCACAACCTGAGCGACACCA | P*reaL-*LacZ cloning |
| ReaL-Pro-R  pUCP20-RpoS-F | CGCGGATCCGCACCGGGTCGCGATCAG  TACTCAGAATTCCCCGGGCTTGAGTCGAAC | P*reaL-*LacZ cloning  RpoS overexpression |
| pUCP20-RpoS-R | ACTCAAAGCTTTCACTGGAACAGCGCGTC | RpoS overexpression |
| pUCP20-KatA-F | TGCGAATTCGCACGTTAGCCGTTGAGGAG | KatA overexpression |
| pUCP20-KatA-R  P*_BAD_*-*rpoS*-F3 | CCCGGATCCCTGGCGACGCTGGATCTGT  CCGCTCGAGCTGGCTGCGTCTGGTGGGA | KatA overexpression  RpoS-Flag cloning |
| P*_BAD_*-*rpoS*-F2 | CCGCTCGAGTCGCCGCCAGGGTAAGCC | RpoS-Flag cloning |
| P*_BAD_*-*rpoS*-F1 | CCGCTCGAGTCGAACTCATGCAAGGGATAACGA | RpoS-Flag cloning |
| P*_BAD_*-*rpoS*-RBS-F | CCGGAATTCTTTAACTTTAAGAAGGAGATATACCATGGCACTCAAAAAAGAAGGG | RpoS-Flag cloning |
| P*_BAD_*-*rpoS*-Flag-R | CCCAAGCTTTCACTTGTCGTCGTCGTCCTTGTAGTCCTGGAACAGCGCGTCACTC | RpoS-Flag cloning |
| pET28a-RpoS-His-F  pET28a-RpoS-His-R | TACTCAGAATTCGCACTCAAAAAAGAAGGGCC  CCCAAGCTTCAAGAGCAGACACAAAAAACCC | RpoS -His cloning  RpoS -His cloning |
| ReaL-T7-F | AAATTAATACGACTCACTATAGTCCATCCAGCGCTGTACTATCC | ReaL transcription |
| ReaL-T7-R | GGCGCGAGAGGAGCAAAA | ReaL transcription |
| *rpoS*-Pro-F | GCCTGCGAGCGGTAGTCTGA | EMSA |
| *rpoS*-Pro-R | ACTCGTCCAGCATGATGCCG | EMSA |
| *rpoS*-In-F | CAACTCCAAAAGCCACCACT | EMSA |
| *rpoS*-In-R | TCGAGCTTGTGGGTCAGTTC | EMSA |
| PA1805-RT-F | ATCAGTCTCAATGAAGTC | RT-PCR |
| PA1805-RT-R  RpsL-RT-F  RpsL-RT-R | CATGGATGGATCGAAATC  GTAAGGTATGCCGTGTACG  CACTACGCTGTGCTCTTG | RT-PCR  RT-PCR  RT-PCR |
| RpoS-RT-F | GGACTCGGACAAGACCCTG | RT-PCR |
| RpoS-RT-R  KatA-RT-F  KatA-RT-R  KatB-RT-F  KatB-RT-R  AhpB-RT-F | GCCACTGGTCGATGCTTT AAGAGCTATCGGCACATC TGGAACTTGACCCAGAAG  TACAGCCACATGACCAAT  CTTGAGCACCTGGATGTA  CGTCGTGCTGTTCTTCTG | RT-PCR  RT-PCR  RT-PCR  RT-PCR  RT-PCR  RT-PCR |
| AhpB-RT-F  AhpC-RT-F  AhpC-RT-R  OxyR-RT-F  OxyR-RT-R | TTATCCATGCGGTTGTTGT  AAGTGGTCGGTCCTGAT  TGTTGGCGGCGTCTT  GCTGCTCAACGACAAGA  ATGTGGCGGATGGTCTC | RT-PCR  RT-PCR  RT-PCR  RT-PCR  RT-PCR |
| RecG-RT-F  RecG-RT-R  ReaL-RT-F  ReaL-RT-F | CCTACTGGGTGTGTAC  AGGAAAGCTCTTCGTA  AGCGCTGTACTATCCCTTCC  CCGGAGGTGCGGATCT | RT-PCR  RT-PCR  RT-PCR  RT-PCR |
| RgsA-RT-F | GAACATGACCAATACTGA | RT-PCR |
| RgsA-RT-R | ATTAGGAGTGGAACAAAG | RT-PCR |
| 16S-S-F  16S-S-R  16S-M-F  16S-M-R  16S-T-F  16S-T-R | TGAGCCAAGTTTAGGGTTTTCT  CCGCTCGACTTGCATGTGT  GGCGACCACCTGGACTGATA  ACATCGTTTACGGCGTGGAC  CTGGGGTGAAGTCGTAACAAG  TGTGGGAGCTTATGAAGAAGC | RT-PCR  RT-PCR  RT-PCR  RT-PCR  RT-PCR  RT-PCR |

The enzymes sites are indicated by the underline

GEN^r^, gentamycin resistance; AMP^r^, ampicillin resistance; TET^r^, tetracycline resistance; CAR^r^, carbenicilin resistance; STR^r^, streptomycin resistance; SPT^r^, spectinomycin resistance; KAN^r^, kanamycin resistance;

**References**

1. Liberati, N.T., Urbach, J.M., Miyata, S., Lee, D.G., Drenkard, E., Wu, G., Villanueva, J., Wei, T. and Ausubel, F.M. (2006) An ordered, nonredundant library of *Pseudomonas aeruginosa* strain PA14 transposon insertion mutants. *Proc Natl Acad Sci U S A*, **103**, 2833-2838.

2. Choi, K.-H. and Schweizer, H.P. (2006) mini-Tn7 insertion in bacteria with single attTn7 sites: example *Pseudomonas aeruginosa*. *Nat Protoc*, **1**, 153-161.

3. Fürste, J.P., Pansegrau, W., Frank, R., Blöcker, H., Scholz, P., Bagdasarian, M. and Lanka, E. (1986) Molecular cloning of the plasmid RP4 primase region in a multi-host-range tacP expression vector. *Gene*, **48**, 119-131.

4. Liang, R. and Liu, J. (2010) Scarless and sequential gene modification in *Pseudomonas* using PCR product flanked by short homology regions. *BMC Microbiol*, **10**, 209.

5. Weng, Y., Chen, F., Liu, Y., Zhao, Q., Chen, R., Pan, X., Liu, C., Cheng, Z., Jin, S., Jin, Y. *et al.* (2016) *Pseudomonas aeruginosa* Enolase Influences Bacterial Tolerance to Oxidative Stresses and Virulence. *Front Microbiol*, **7**, 1999.
