# Supplementary material for: Endoribonuclease YbeY Is Essential for RNA Processing and Virulence in Pseudomonas aeruginosa
Source: mBio. 2020 Jun 30;11(3):e00659-20. doi: 10.1128/mBio.00659-20 (PMC7327168; doi:10.1128/mBio.00659-20)
Supplement: TABLE S2 [file mBio.00659-20-st002.docx]

**Supplemental Material**

**Table S2. The RNA-seq results of Δ*ybeY* and PA14.**

Genes that displayed similar expression patterns in the *ybeY* and *rpoS* mutants in comparison to the wild type strain are shown in red, and those with opposite expression patterns are shown in blue.

| **Locus-tag** | **Gene name** | **Product** | **Fold change**  **Δ*ybeY*/PA14** | | **P-Val** |
| --- | --- | --- | --- | --- | --- |
| PA0044 | *exoT* | exoenzyme T | | 12.29 | 9.92E-06 |
| PA0105 | *coxB* | cytochrome C oxidase subunit II | | 0.21 | 8.60E-05 |
| PA0111 | PA0111 | hypothetical protein | | 0.12 | 5.29E-06 |
| PA0116 | PA0116 | hypothetical protein | | 7.61 | 4.29E-04 |
| PA0119 | PA0119 | C4-dicarboxylate transporter DctA | | 6.50 | 1.44E-03 |
| PA0139 | *ahpC* | alkyl hydroperoxide reductase | | 0.03 | 3.06E-12 |
| PA0173 | PA0173 | chemotaxis response regulator protein-glutamate methylesterase | | 0.27 | 8.10E-04 |
| PA0175 | PA0175 | chemotaxis protein methyltransferase | | 0.21 | 9.91E-05 |
| PA0176 | *aer2* | aerotaxis transducer Aer2 | | 0.20 | 7.04E-05 |
| PA0177 | PA0177 | purine-binding chemotaxis protein | | 0.17 | 2.16E-05 |
| PA0178 | PA0178 | two-component sensor | | 0.29 | 8.55E-04 |
| PA0179 | PA0179 | two-component response regulator | | 0.31 | 1.31E-03 |
| PA0180 | *cttP* | trichloroethylene chemotactic transducer CttP | | 0.27 | 5.34E-04 |
| PA0201 | PA0201 | hypothetical protein | | 11.06 | 3.33E-05 |
| PA0208 | *mdcA* | malonate decarboxylase subunit alpha | | 0.25 | 3.53E-04 |
| PA0209 | PA0209 | 2-(5''-triphosphoribosyl)-3'-dephosphocoenzyme-A synthase | | 0.12 | 9.02E-07 |
| PA0210 | *mdcC* | malonate decarboxylase acyl carrier protein | | 0.07 | 7.49E-07 |
| PA0211 | *mdcD* | malonate decarboxylase subunit beta | | 0.13 | 2.99E-06 |
| PA0212 | *mdcE* | malonate decarboxylase subunit gamma | | 0.23 | 2.22E-04 |
| PA0249 | PA0249 | acetyltransferase | | 0.10 | 3.10E-07 |
| PA0250 | PA0250 | hypothetical protein | | 0.12 | 1.02E-06 |
| PA0329 | PA0329 | hypothetical protein | | 0.34 | 2.75E-03 |
| PA0356 | PA0356 | hypothetical protein | | 6.18 | 1.89E-03 |
| PA0423 | *pasP* | hypothetical protein | | 0.27 | 5.90E-04 |
| PA0442 | PA0442 | hypothetical protein | | 46.73 | 6.91E-05 |
| PA0449 | PA0449 | acyl-CoA thioesterase | | 0.10 | 2.66E-07 |
| PA0463 | *creB* | DNA-binding response regulator CreB | | 6.41 | 1.52E-03 |
| PA0471 | PA0471 | transmembrane sensor | | 0.31 | 1.69E-03 |
| PA0472 | PA0472 | RNA polymerase sigma factor | | 0.30 | 1.28E-03 |
| PA0518 | *nirM* | cytochrome C-551 | | 0.30 | 1.19E-03 |
| PA0519 | *nirS* | nitrite reductase | | 0.25 | 3.48E-04 |
| PA0520 | *nirQ* | denitrification regulatory protein NirQ | | 0.19 | 4.79E-05 |
| PA0521 | PA0521 | cytochrome C oxidase subunit | | 0.22 | 1.59E-04 |
| PA0522 | PA0522 | hypothetical protein | | 0.26 | 9.96E-04 |
| PA0523 | *norC* | nitric oxide reductase subunit C | | 0.05 | 2.01E-10 |
| PA0524 | *norB* | nitric oxide reductase subunit B | | 0.07 | 9.94E-09 |
| PA0525 | PA0525 | denitrification protein NorD | | 0.26 | 3.83E-04 |
| PA0526 | PA0526 | hypothetical protein | | 0.33 | 2.44E-03 |
| PA0545 | PA0545 | hypothetical protein | | 5.89 | 2.38E-03 |
| PA0572 | PA0572 | hypothetical protein | | 0.18 | 2.19E-05 |
| PA0586 | PA0586 | hypothetical protein | | 0.31 | 1.42E-03 |
| PA0587 | PA0587 | hypothetical protein | | 0.31 | 1.38E-03 |
| PA0588 | PA0588 | hypothetical protein | | 0.32 | 1.66E-03 |
| PA0713 | PA0713 | hypothetical protein | | 0.32 | 2.05E-03 |
| PA0718 | PA0718 | hypothetical protein | | 40.30 | 1.95E-04 |
| PA0734 | PA0734 | hypothetical protein | | 13.44 | 4.36E-04 |
| PA0744 | PA0744 | enoyl-CoA hydratase | | 0.33 | 2.42E-03 |
| PA0745 | PA0745 | enoyl-CoA hydratase | | 0.30 | 1.27E-03 |
| PA0761 | *nadB* | L-aspartate oxidase | | 6.69 | 9.75E-04 |
| PA0836 | *ackA* | acetate kinase | | 6.83 | 8.47E-04 |
| PA0845 | PA0845 | neutral ceramidase | | 7.99 | 2.93E-04 |
| PA0848 | PA0848 | alkyl hydroperoxide reductase | | 0.01 | 3.04E-15 |
| PA0849 | *trxB2* | thioredoxin reductase | | 0.12 | 9.00E-07 |
| PA0852 | *cbpD* | chitin-binding protein CbpD | | 0.27 | 5.07E-04 |
| PA0858 | PA0858 | hypothetical protein | | 0.19 | 3.81E-05 |
| PA0865 | *hpd* | 4-hydroxyphenylpyruvate dioxygenase | | 0.28 | 7.42E-04 |
| PA0867 | *mliC* | lysozyme inhibitor | | 0.33 | 2.36E-03 |
| PA0870 | *phhC* | aromatic amino acid aminotransferase | | 0.33 | 1.98E-03 |
| PA0871 | *phhB* | pterin-4-alpha-carbinolamine dehydratase | | 0.19 | 4.37E-05 |
| PA0888 | *aotJ* | arginine/ornithine ABC transporter substrate-binding protein AotJ | | 0.28 | 7.09E-04 |
| PA0889 | *aotQ* | arginine/ornithine ABC transporter permease AotQ | | 0.32 | 2.16E-03 |
| PA0913 | *mgtE* | Mg transporter MgtE | | 8.12 | 2.56E-04 |
| PA0942 | PA0942 | transcriptional regulator | | 6.43 | 1.32E-03 |
| PA0962 | PA0962 | DNA-binding stress protein | | 0.20 | 4.86E-05 |
| PA1041 | PA1041 | hypothetical protein | | 0.18 | 2.11E-05 |
| PA1051 | PA1051 | transporter | | 6.89 | 1.17E-03 |
| PA1155 | *nrdB* | ribonucleotide-diphosphate reductase subunit beta | | 0.33 | 2.38E-03 |
| PA1190 | PA1190 | hypothetical protein | | 0.21 | 1.14E-04 |
| PA1202 | PA1202 | hydrolase | | 0.18 | 2.73E-05 |
| PA1215 | PA1215 | hypothetical protein | | 0.27 | 6.77E-04 |
| PA1216 | PA1216 | hypothetical protein | | 0.28 | 8.31E-04 |
| PA1248 | *aprF* | alkaline protease secretion protein AprF | | 0.25 | 3.51E-04 |
| PA1249 | *aprA* | alkaline metalloproteinase | | 0.03 | 1.18E-12 |
| PA1337 | *ansB* | glutaminase-asparaginase | | 0.19 | 3.82E-05 |
| PA1344 | PA1344 | short-chain dehydrogenase | | 0.33 | 2.16E-03 |
| PA1353 | PA1353 | hypothetical protein | | 0.31 | 2.04E-03 |
| PA1416 | PA1416 | hypothetical protein | | 7.46 | 5.11E-04 |
| PA1417 | PA1417 | hypothetical protein | | 7.78 | 5.47E-04 |
| PA1429 | PA1429 | cation-transporting P-type ATPase | | 6.09 | 1.84E-03 |
| PA1432 | *lasI* | acyl-homoserine-lactone synthase | | 0.21 | 1.00E-04 |
| PA1541 | PA1541 | drug efflux transporter | | 35.71 | 1.28E-09 |
| PA1559 | PA1559 | _ | | 6.57 | 1.17E-03 |
| PA1568 | PA1568 | hypothetical protein | | 17.69 | 2.69E-05 |
| PA1588 | *sucC* | succinyl-CoA ligase subunit beta | | 0.33 | 1.97E-03 |
| PA1604 | PA1604 | hypothetical protein | | 8.67 | 1.71E-04 |
| PA1673 | PA1673 | bacteriohemerythrin | | 9.88 | 5.57E-05 |
| PA1690 | *pscU* | translocation protein in type III secretion | | 7.69 | 4.87E-04 |
| PA1691 | *pscT* | translocation protein in type III secretion | | 9.51 | 2.74E-04 |
| PA1694 | *pscQ* | type III secretion system protein | | 7.42 | 7.00E-04 |
| PA1695 | *pscP* | translocation protein in type III secretion | | 7.23 | 1.13E-03 |
| PA1696 | *pscO* | translocation protein in type III secretion | | 9.97 | 1.92E-04 |
| PA1697 | PA1697 | type III secretion system ATPase | | 30.51 | 5.38E-09 |
| PA1698 | *popN* | type III secretion outer membrane protein PopN | | 18.05 | 5.95E-07 |
| PA1699 | PA1699 | hypothetical protein | | 122.33 | 4.81E-08 |
| PA1700 | PA1700 | hypothetical protein | | 23.19 | 1.18E-06 |
| PA1702 | PA1702 | hypothetical protein | | 22.89 | 5.75E-06 |
| PA1703 | *pcrD* | type III secretory apparatus protein PcrD | | 10.92 | 2.73E-05 |
| PA1704 | *pcrR* | transcriptional regulator PcrR | | 9.00 | 3.34E-04 |
| PA1705 | *pcrG* | type III secretion regulator | | 17.92 | 8.15E-07 |
| PA1706 | *pcrV* | type III secretion protein PcrV | | 16.06 | 1.12E-06 |
| PA1707 | *pcrH* | regulatory protein PcrH | | 14.89 | 2.40E-06 |
| PA1708 | *popB* | translocator protein PopB | | 19.11 | 2.33E-07 |
| PA1709 | *popD* | translocator outer membrane protein PopD | | 18.83 | 2.66E-07 |
| PA1710 | *exsC* | exoenzyme S synthesis protein ExsC | | 9.97 | 5.26E-05 |
| PA1711 | PA1711 | hypothetical protein | | 9.08 | 2.08E-04 |
| PA1712 | *exsB* | exoenzyme S synthesis protein ExsB | | 8.42 | 1.99E-04 |
| PA1713 | *exsA* | exoenzyme S transcriptional regulator ExsA | | 9.88 | 5.66E-05 |
| PA1714 | *exsD* | hypothetical protein | | 17.14 | 6.23E-07 |
| PA1715 | *pscB* | type III export apparatus protein | | 19.88 | 2.56E-07 |
| PA1716 | *pscC* | type III secretion outer membrane protein PscC | | 18.46 | 3.55E-07 |
| PA1717 | *pscD* | type III export protein PscD | | 15.43 | 1.84E-06 |
| PA1719 | *pscF* | type III export protein PscF | | 11.99 | 1.58E-04 |
| PA1720 | *pscG* | type III export protein PscG | | 22.91 | 1.54E-07 |
| PA1721 | *pscH* | type III export protein PscH | | 20.20 | 8.87E-07 |
| PA1722 | *pscI* | type III export protein PscI | | 12.57 | 2.24E-05 |
| PA1723 | *pscJ* | type III export protein PscJ | | 22.89 | 8.14E-08 |
| PA1724 | *pscK* | type III export protein PscK | | 16.71 | 1.61E-06 |
| PA1725 | *pscL* | type III secretion system protein | | 13.19 | 8.27E-06 |
| PA1728 | PA1728 | hypothetical protein | | 0.18 | 2.86E-05 |
| PA1784 | PA1784 | hypothetical protein | | 0.24 | 2.25E-04 |
| PA1830 | PA1830 | hypothetical protein | | 0.29 | 9.88E-04 |
| PA1871 | *lasA* | protease LasA | | 0.05 | 2.15E-10 |
| PA1883 | PA1883 | NADH-quinone oxidoreductase subunit A | | 10.20 | 2.48E-03 |
| PA1900 | *phzB2* | phenazine biosynthesis protein PhzB | | 0.23 | 1.91E-04 |
| PA1914 | PA1914 | hypothetical protein | | 0.21 | 8.86E-05 |
| PA1985 | *pqqA* | coenzyme PQQ synthesis protein A | | 0.28 | 1.02E-03 |
| PA2031 | PA2031 | hypothetical protein | | 0.30 | 1.40E-03 |
| PA2081 | *kynB* | kynurenine formamidase KynB | | 5.98 | 2.41E-03 |
| PA2093 | PA2093 | RNA polymerase sigma factor | | 8.05 | 5.80E-04 |
| PA2114 | PA2114 | major facilitator superfamily transporter | | 8.05 | 3.31E-04 |
| PA2125 | PA2125 | aldehyde dehydrogenase | | 7.26 | 6.81E-04 |
| PA2126 | PA2126 | hypothetical protein | | 13.39 | 6.44E-06 |
| PA2126 | PA2126 | hypothetical protein | | 15.74 | 2.05E-06 |
| PA2127 | PA2127 | hypothetical protein | | 17.78 | 4.38E-07 |
| PA2134 | PA2134 | hypothetical protein | | 0.22 | 1.72E-04 |
| PA2136 | PA2136 | hypothetical protein | | 7.63 | 5.13E-04 |
| PA2146 | PA2146 | hypothetical protein | | 0.03 | 1.61E-12 |
| PA2159 | PA2159 | hypothetical protein | | 0.19 | 4.97E-05 |
| PA2166 | PA2166 | hypothetical protein | | 0.19 | 4.14E-05 |
| PA2169 | PA2169 | hypothetical protein | | 0.11 | 4.11E-07 |
| PA2171 | PA2171 | hypothetical protein | | 0.13 | 2.48E-06 |
| PA2172 | PA2172 | hypothetical protein | | 0.29 | 1.00E-03 |
| PA2173 | PA2173 | hypothetical protein | | 0.11 | 9.49E-07 |
| PA2189 | PA2189 | hypothetical protein | | 6.51 | 1.33E-03 |
| PA2193 | *hcnA* | hydrogen cyanide synthase subunit HcnA | | 11.73 | 1.54E-05 |
| PA2194 | *hcnB* | hydrogen cyanide synthase subunit HcnB | | 6.79 | 8.94E-04 |
| PA2196 | PA2196 | transcriptional regulator | | 6.67 | 1.04E-03 |
| PA2300 | *chiC* | chitinase | | 0.25 | 3.36E-04 |
| PA2433 | PA2433 | hypothetical protein | | 0.30 | 1.09E-03 |
| PA2453 | PA2453 | hypothetical protein | | 6.75 | 1.26E-03 |
| PA2479 | PA2479 | two-component response regulator | | 9.72 | 8.76E-05 |
| PA2500 | PA2500 | major facilitator superfamily transporter | | 7.45 | 4.65E-04 |
| PA2566 | PA2566 | hypothetical protein | | 0.16 | 1.05E-05 |
| PA2567 | PA2567 | hypothetical protein | | 6.50 | 1.26E-03 |
| PA2570 | *lecA* | PA-I galactophilic lectin | | 0.08 | 3.32E-08 |
| PA2588 | PA2588 | transcriptional regulator | | 0.31 | 1.43E-03 |
| PA2604 | PA2604 | hypothetical protein | | 0.34 | 2.61E-03 |
| PA2659 | PA2659 | hypothetical protein | | 0.32 | 2.10E-03 |
| PA2711 | PA2711 | spermidine/putrescine-binding protein | | 5.90 | 2.51E-03 |
| PA2753 | PA2753 | hypothetical protein | | 6.38 | 1.36E-03 |
| PA2759 | PA2759 | hypothetical protein | | 7.16 | 6.60E-04 |
| PA2864 | PA2864 | hypothetical protein | | 0.19 | 5.74E-05 |
| PA2867 | PA2867 | chemotaxis transducer | | 7.59 | 4.02E-04 |
| PA2868 | PA2868 | hypothetical protein | | 0.15 | 8.14E-06 |
| PA2919 | PA2919 | hypothetical protein | | 0.22 | 9.97E-04 |
| PA2937 | PA2937 | hypothetical protein | | 0.23 | 4.36E-04 |
| PA2939 | PA2939 | aminopeptidase | | 0.17 | 1.51E-05 |
| PA2970 | *rpmF* | 50S ribosomal protein L32 | | 0.28 | 8.73E-04 |
| PA3032 | *snr1* | cytochrome C Snr1 | | 0.04 | 1.70E-11 |
| PA3049 | *rmf* | ribosome modulation factor | | 0.04 | 2.02E-11 |
| PA3057 | PA3057 | hypothetical protein | | 8.31 | 7.44E-04 |
| PA3126 | *ibpA* | heat-shock protein IbpA | | 0.18 | 2.21E-05 |
| PA3231 | PA3231 | hypothetical protein | | 0.29 | 1.01E-03 |
| PA3237 | PA3237 | hypothetical protein | | 0.18 | 3.73E-05 |
| PA3278 | PA3278 | hypothetical protein | | 9.91 | 6.15E-05 |
| PA3282 | PA3282 | hypothetical protein | | 8.43 | 4.14E-04 |
| PA3283 | PA3283 | hypothetical protein | | 8.97 | 1.83E-04 |
| PA3284 | PA3284 | hypothetical protein | | 6.40 | 1.70E-03 |
| PA3287 | PA3287 | hypothetical protein | | 0.04 | 3.40E-11 |
| PA3288 | PA3288 | hypothetical protein | | 0.24 | 2.39E-04 |
| PA3327 | PA3327 | non-ribosomal peptide synthetase | | 6.84 | 8.45E-04 |
| PA3328 | PA3328 | FAD-dependent monooxygenase | | 14.41 | 3.42E-06 |
| PA3329 | PA3329 | hypothetical protein | | 9.88 | 6.40E-05 |
| PA3330 | PA3330 | short-chain dehydrogenase | | 8.32 | 2.31E-04 |
| PA3331 | PA3331 | cytochrome P450 | | 6.69 | 1.05E-03 |
| PA3332 | PA3332 | hypothetical protein | | 9.32 | 1.57E-04 |
| PA3333 | *fabH2* | 3-oxoacyl-ACP synthase III | | 5.97 | 2.31E-03 |
| PA3336 | PA3336 | major facilitator superfamily transporter | | 15.50 | 1.59E-06 |
| PA3337 | *rfaD* | ADP-L-glycero-D-mannoheptose-6-epimerase | | 11.36 | 1.86E-05 |
| PA3397 | *fpr* | ferredoxin-NADP reductase | | 0.18 | 3.19E-05 |
| PA3414 | PA3414 | hypothetical protein | | 0.33 | 2.56E-03 |
| PA3415 | PA3415 | branched-chain alpha-keto acid dehydrogenase subunit E2 | | 0.23 | 1.63E-04 |
| PA3416 | PA3416 | pyruvate dehydrogenase E1 component subunit beta | | 0.25 | 3.78E-04 |
| PA3451 | PA3451 | hypothetical protein | | 0.24 | 3.09E-04 |
| PA3478 | *rhlB* | rhamnosyltransferase subunit B | | 0.33 | 2.12E-03 |
| PA3496 | PA3496 | hypothetical protein | | 0.22 | 1.71E-04 |
| PA3518 | PA3518 | hypothetical protein | | 10.22 | 7.21E-05 |
| PA3519 | PA3519 | hypothetical protein | | 16.48 | 8.84E-07 |
| PA3521 | PA3521 | hypothetical protein | | 6.14 | 2.39E-03 |
| PA3522 | PA3522 | resistance-nodulation-cell division (RND) efflux transporter | | 9.37 | 9.21E-05 |
| PA3523 | PA3523 | resistance-nodulation-cell division (RND) efflux membrane fusion protein | | 25.01 | 3.30E-08 |
| PA3530 | PA3530 | hypothetical protein | | 0.31 | 1.56E-03 |
| PA3533 | PA3533 | hypothetical protein | | 0.22 | 1.38E-04 |
| PA3569 | *mmsB* | 3-hydroxyisobutyrate dehydrogenase | | 0.19 | 5.31E-05 |
| PA3572 | PA3572 | hypothetical protein | | 6.37 | 1.48E-03 |
| PA3573 | PA3573 | major facilitator superfamily transporter | | 14.72 | 2.52E-06 |
| PA3574 | *nalD* | transcriptional regulator | | 16.68 | 8.18E-07 |
| PA3574a | PA3574a | copper chaperone CopZ | | 27.85 | 1.07E-08 |
| PA3613 | PA3613 | hypothetical protein | | 7.54 | 4.22E-04 |
| PA3622 | *rpoS* | RNA polymerase sigma factor RpoS | | 0.34 | 2.75E-03 |
| PA3655 | *tsf* | elongation factor Ts | | 0.34 | 2.52E-03 |
| PA3723 | PA3723 | FMN oxidoreductase | | 0.32 | 1.62E-03 |
| PA3724 | *lasB* | elastase LasB | | 0.12 | 1.14E-06 |
| PA3813 | *iscU* | scaffold protein | | 0.23 | 1.75E-04 |
| PA3814 | *iscS* | cysteine desulfurase | | 0.24 | 2.45E-04 |
| PA3839 | PA3839 | sodium:sulfate symporter | | 5.87 | 2.41E-03 |
| PA3871 | PA3871 | PpiC-type peptidyl-prolyl cis-trans isomerase | | 6.49 | 1.60E-03 |
| PA3874 | *narH* | respiratory nitrate reductase subunit beta | | 7.32 | 6.13E-04 |
| PA3875 | *narG* | respiratory nitrate reductase subunit alpha | | 15.04 | 1.95E-06 |
| PA3876 | *narK2* | nitrite extrusion protein 2 | | 36.08 | 1.32E-09 |
| PA3877 | *narK1* | nitrite extrusion protein 1 | | 53.16 | 2.57E-11 |
| PA3879 | *narL* | transcriptional regulator NarL | | 7.16 | 6.54E-04 |
| PA3914 | *moeA1* | molybdenum cofactor biosynthesis protein A | | 33.46 | 2.04E-09 |
| PA3915 | *moaB1* | molybdopterin biosynthesis protein B | | 37.79 | 7.97E-10 |
| PA3920 | PA3920 | metal transporting P-type ATPase | | 20.09 | 1.49E-07 |
| PA3922 | PA3922 | hypothetical protein | | 0.22 | 1.29E-04 |
| PA3930 | *cioA* | cyanide insensitive terminal oxidase | | 0.30 | 1.07E-03 |
| PA3968 | PA3968 | pseudouridine synthase | | 9.17 | 1.25E-04 |
| PA3982 | PA3982 | metalloprotease | | 0.00 | 7.91E-14 |
| PA3986 | PA3986 | hypothetical protein | | 0.25 | 3.16E-04 |
| PA4078 | PA4078 | nonribosomal peptide synthetase | | 0.28 | 7.89E-04 |
| PA4139 | PA4139 | hypothetical protein | | 0.16 | 9.89E-06 |
| PA4141 | PA4141 | hypothetical protein | | 0.28 | 6.36E-04 |
| PA4171 | PA4171 | protease | | 0.31 | 2.05E-03 |
| PA4175 | *piv* | endopeptidase IV | | 0.20 | 4.99E-05 |
| PA4206 | *mexH* | resistance-nodulation-cell division (RND) efflux membrane fusion protein | | 0.27 | 5.35E-04 |
| PA4207 | *mexI* | resistance-nodulation-cell division (RND) efflux transporter | | 0.25 | 3.20E-04 |
| PA4208 | *opmD* | hypothetical protein | | 0.32 | 1.96E-03 |
| PA4218 | PA4218 | transporter | | 0.33 | 2.25E-03 |
| PA4219 | PA4219 | hypothetical protein | | 0.25 | 3.46E-04 |
| PA4220 | PA4220 | hypothetical protein | | 0.07 | 4.63E-07 |
| PA4221 | *fptA* | Fe(III)-pyochelin outer membrane receptor | | 0.09 | 5.54E-08 |
| PA4222 | PA4222 | ABC transporter ATP-binding protein | | 0.12 | 7.63E-07 |
| PA4223 | PA4223 | ABC transporter ATP-binding protein | | 0.17 | 1.69E-05 |
| PA4224 | *pchG* | pyochelin biosynthetic protein PchG | | 0.13 | 2.74E-06 |
| PA4225 | *pchF* | pyochelin synthetase | | 0.16 | 9.45E-06 |
| PA4226 | *pchE* | dihydroaeruginoic acid synthetase | | 0.11 | 3.03E-07 |
| PA4227 | *pchR* | transcriptional regulator PchR | | 0.29 | 1.11E-03 |
| PA4228 | *pchD* | 2%2C3-dihydroxybenzoate-AMP ligase | | 0.05 | 2.06E-10 |
| PA4229 | *pchC* | pyochelin biosynthetic protein PchC | | 0.07 | 2.07E-08 |
| PA4230 | *pchB* | isochorismate-pyruvate lyase | | 0.05 | 2.58E-09 |
| PA4231 | *pchA* | salicylate biosynthesis isochorismate synthase | | 0.08 | 2.60E-08 |
| PA4236 | *katA* | catalase | | 0.03 | 9.11E-12 |
| PA4293 | *pprA* | two-component sensor PprA | | 0.32 | 1.99E-03 |
| PA4296 | *pprB* | two-component response regulator PprB | | 0.32 | 1.77E-03 |
| PA4298 | PA4298 | hypothetical protein | | 0.16 | 5.51E-05 |
| PA4299 | *tadD* | type II secretion system protein TadD | | 0.20 | 8.83E-05 |
| PA4301 | *tadB* | type II secretion system protein TadB | | 0.18 | 3.93E-05 |
| PA4302 | *tadA* | ATPase TadA | | 0.27 | 5.76E-04 |
| PA4304 | *rcpA* | type II/III secretion system protein | | 0.20 | 7.09E-05 |
| PA4305 | *rcpC* | hypothetical protein | | 0.24 | 2.56E-04 |
| PA4306 | *flp* | type IVb pilin Flp | | 0.03 | 9.00E-13 |
| PA4318 | PA4318 | hypothetical protein | | 10.53 | 4.26E-05 |
| PA4328 | PA4328 | hypothetical protein | | 6.06 | 1.93E-03 |
| PA4344 | PA4344 | hydrolase | | 12.41 | 9.89E-06 |
| PA4360a | PA4360a | hypothetical protein | | 10.82 | 6.40E-04 |
| PA4366 | *sodB* | superoxide dismutase | | 0.28 | 7.76E-04 |
| PA4377 | PA4377 | hypothetical protein | | 0.22 | 1.49E-04 |
| PA4386 | *groES* | co-chaperonin GroES | | 0.23 | 1.51E-04 |
| PA4387 | PA4387 | phage exclusion suppressor FxsA | | 0.26 | 3.94E-04 |
| PA4563 | *rpsT* | 30S ribosomal protein S20 | | 0.19 | 4.55E-05 |
| PA4567 | *rpmA* | 50S ribosomal protein L27 | | 0.33 | 2.11E-03 |
| PA4570 | PA4570 | hypothetical protein | | 0.19 | 7.18E-05 |
| PA4571 | PA4571 | cytochrome C | | 8.95 | 1.23E-04 |
| PA4573 | PA4573 | hypothetical protein | | 0.29 | 9.26E-04 |
| PA4590 | *pra* | protein activator | | 0.26 | 4.61E-04 |
| PA4596 | PA4596 | transcriptional regulator | | 9.65 | 7.53E-05 |
| PA4605 | PA4605 | hypothetical protein | | 0.22 | 2.34E-04 |
| PA4607 | PA4607 | hypothetical protein | | 0.06 | 3.14E-09 |
| PA4612 | PA4612 | hypothetical protein | | 0.12 | 2.08E-06 |
| PA4613 | *katB* | catalase | | 0.11 | 3.31E-07 |
| PA4637a | PA4637a | hypothetical protein | | 0.08 | 5.44E-08 |
| PA4641 | PA4641 | _ | | 0.29 | 1.15E-03 |
| PA4738 | PA4738 | hypothetical protein | | 0.14 | 2.61E-06 |
| PA4739 | PA4739 | hypothetical protein | | 0.12 | 1.01E-06 |
| PA4764 | *fur* | ferric uptake regulation protein | | 0.28 | 6.38E-04 |
| PA4817 | PA4817 | hypothetical protein | | 14.74 | 4.59E-04 |
| PA4874 | PA4874 | hypothetical protein | | 0.24 | 2.76E-04 |
| PA4878 | PA4878 | transcriptional regulator | | 6.81 | 8.99E-04 |
| PA4935 | *rpsF* | 30S ribosomal protein S6 | | 0.25 | 2.89E-04 |
| PA4985 | PA4985 | hypothetical protein | | 17.37 | 7.61E-07 |
| PA5053 | *hslV* | ATP-dependent protease peptidase subunit | | 0.25 | 3.99E-04 |
| PA5054 | *hslU* | ATP-dependent protease ATP-binding subunit HslU | | 0.32 | 1.65E-03 |
| PA5059 | PA5059 | transcriptional regulator | | 0.29 | 1.05E-03 |
| PA5061 | PA5061 | hypothetical protein | | 0.13 | 2.41E-06 |
| PA5183a | PA5183a | hypothetical protein | | 5.79 | 2.60E-03 |
| PA5217 | PA5217 | iron ABC transporter substrate-binding protein | | 0.23 | 1.70E-04 |
| PA5232 | PA5232 | hypothetical protein | | 7.63 | 3.83E-04 |
| PA5240 | *trxA* | thioredoxin | | 0.21 | 7.63E-05 |
| PA5271 | PA5271 | hypothetical protein | | 0.25 | 3.05E-04 |
| PA5285 | PA5285 | hypothetical protein | | 0.31 | 1.53E-03 |
| PA5315 | *rpmG* | 50S ribosomal protein L33 | | 0.17 | 7.38E-05 |
| PA5354 | *glcE* | glycolate oxidase FAD binding subunit | | 0.23 | 2.84E-04 |
| PA5373 | *betB* | betaine aldehyde dehydrogenase | | 0.16 | 1.09E-05 |
| PA5374 | *betI* | BetI family transcriptional regulator | | 0.11 | 4.36E-07 |
| PA5382 | PA5382 | transcriptional regulator | | 7.31 | 6.93E-04 |
| PA5424 | PA5424 | hypothetical protein | | 0.18 | 3.30E-05 |
| PA5440 | PA5440 | peptidase | | 9.22 | 1.05E-04 |
| PA5446 | PA5446 | hypothetical protein | | 0.20 | 5.61E-05 |
| PA5460 | PA5460 | hypothetical protein | | 0.14 | 1.17E-05 |
| PA5461 | PA5461 | hypothetical protein | | 0.25 | 3.31E-04 |
| PA5471 | PA5471 | hypothetical protein | | 6.40 | 1.63E-03 |
| PA5546 | PA5546 | hypothetical protein | | 0.19 | 3.30E-05 |
